# Stereoanalysis of the Antiparasitic Natural Product Callunene and Its Synthetic Intermediates

**DOI:** 10.1021/acs.jnatprod.4c01424

**Published:** 2025-03-03

**Authors:** Jiří Ferenczei, Vilém Blahout, Hana Dvořáková, Andrea Brancale, Petra Cuřínová, Magdaléna Labíková, Michal Kohout, Vladimír Setnička, Pavla Perlíková

**Affiliations:** † Department of Organic Chemistry, Faculty of Chemical Technology, University of Chemistry and Technology Prague, Technická 5, Prague 6 16628, Czech Republic; ‡ Laboratory of Nuclear Magnetic Resonance Spectroscopy, University of Chemistry and Technology Prague, Technická 5, Prague 6 16628, Czech Republic; § Department of Analytical Chemistry, Faculty of Chemical Engineering, University of Chemistry and Technology Prague, Technická 5, Prague 6 16628, Czech Republic; ∥ Institute of Organic Chemistry and Biochemistry, Czech Academy of Sciences, Flemingovo nám. 2, Prague 6 16000, Czech Republic

## Abstract

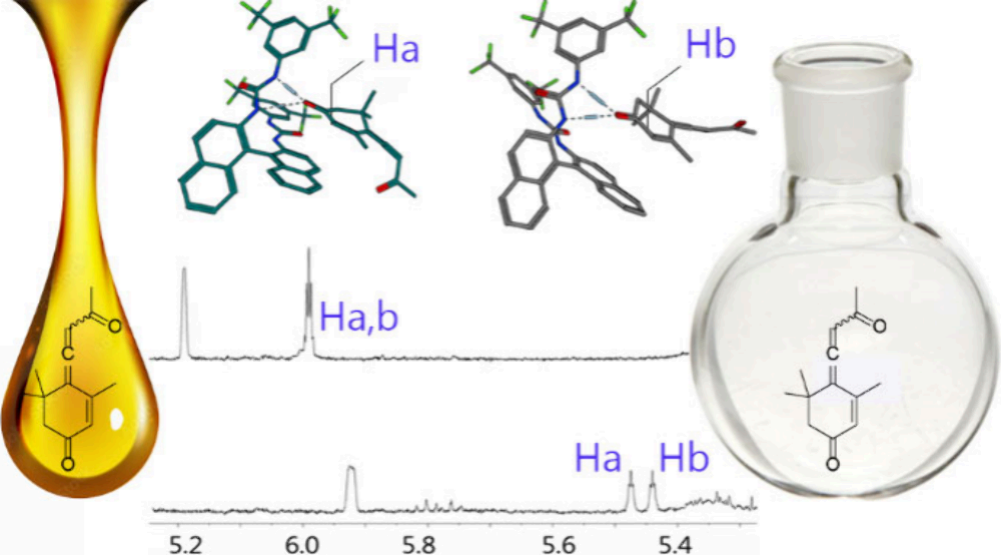

The recent popularity of indoor farming has brought about problems with parasites spreading among pollinator colonies. The natural product callunene (**1**) can be used in the prophylaxis of bumblebees against *Crithidia* infection. Here, we report the synthesis of callunene (**1**), its enantioseparation, and a method for analyzing its optical purity. The approach was applied to determine the configuration of callunene extracted from heather honey. The proposed method is also applicable to the analysis of mixtures of diastereomers obtained during callunene synthesis, which allows the stereospecificity of individual reaction steps to be determined.

Callunene (**1**, 4-(3-oxobut-1-enylidene)-3,5,5-trimethylcyclohex-2-en-1-one or megastigma-4,6,7-trien-3,9-dione) is a natural product present in the nectar of heather (*Calluna vulgaris*) and heather honey^1,2^ or *Vitis vinifera* cv. Sauvignon Blanc juices.^3^ It was recently reported to prevent *Crithidia bombi* infection in bumblebees (*Bombus terrestris*).^1^*Crithidia* is a protozoan of the *Trypanosomatidae* family that affects bumblebees’ ability to distinguish the nectar-containing flowers, leading to starvation and death.^4^ Other reported effects include lower colony reproduction and fitness and impact on foraging behavior and learning abilities.^4^ The antiparasitic effect of callunene (**1**) is based on the removal of the *C. bombi* flagellum, which leads to a reduction in the motility of the parasite and, more importantly, prevents its attachment to the host ileal epithelium.^1^ This effect is prophylactic; therefore, the bumblebees with access to the *Calluna* heather nectar are protected against *C. bombi* infection. On the other hand, the *Calluna* nectar does not have any effect on the bumblebee fitness in ongoing infection. Although heather fields represent an important nutrition source for pollinators, the changes in land uses are leading to heathland loss on a global scale.^5,6^ In this way, bumblebees are losing not only a vital source of food but also a natural remedy essential for their health. While the natural resources are invaluable, isolating and utilizing callunene (**1**) provides an opportunity for preventive measures against *C. bombi* infections, particularly in indoor pollinator colonies. In this regard, ethyl acetate extracts of heather honey, rich in callunene (**1**), were used for feeding in prophylactic studies.^1^

The isolation of callunene (**1**) from natural sources is a challenging process due to its low abundance and the complexity of purification. Additionally, the absolute configuration of callunene (**1**), an axially chiral compound, has not been determined and reported until now. Many allenic natural products, however, exist in plants as single diastereomers, for instance, grasshopper ketone (**2**)^7,8^ or its diastereomer lyratol F (**3**), which differs from the grasshopper ketone (**2**) only in the configuration of the allenic unit.^9^ Knowledge of the absolute configuration and optical purity of biologically active compounds like callunene (**1**) is essential to understand their effects,^10,11^ as well as for their chemical synthesis. Techniques such as chiral gas chromatography (GC),^12^ high-performance liquid chromatography (HPLC),^13,14^ polarimetry, and nuclear magnetic resonance (NMR) spectroscopy^15−17^ can be employed for this purpose. Given the significant potential of callunene (**1**) as a natural antiparasitic agent, this work presents its chemical synthesis and investigates the stereochemistry of callunene (**1**) and its synthetic intermediates, with a focus on approaches for determining their absolute configuration. Particular emphasis is placed on developing methods for obtaining pure enantiomers and advancing the synthetic strategy for callunene (**1**), building on the established reaction sequences.

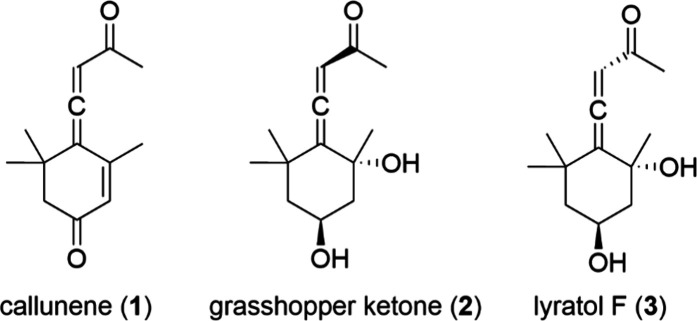



## Results and Discussion

### Synthesis

**Scheme 1:**
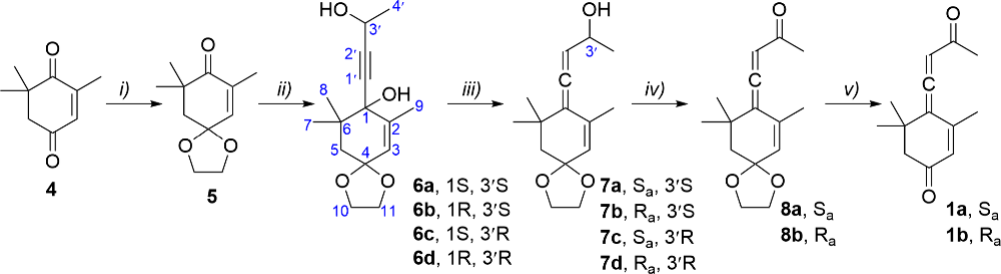
Synthesis of Callunene (**1**) (i) Ethylene glycol, trimethyl orthoformate, TsOH, DCM, reflux, 4 h, 86%; (ii) (1) But-3-yn-2-ol, BuLi, THF, −78 °C 30 min, then (2) **5**, −78 °C to rt 16 h, 48–53%; (iii) LiAlH_4_, Et_2_O, 30 °C, 4 h, 17–18%; (iv) MnO_2_, hexane, rt, 19 h, 67%; (v) PPTS, acetone/water, 60 °C, 1.5 h, 98%.

Most of the steps of the synthesis of callunene (**1**) were already published,^18,19^ but we changed and optimized some of them (Scheme 1). During the synthesis, instability of acetal-protected intermediates **6**–**8** was observed due to spontaneous hydrolysis of the acetal moiety; these intermediates had to be stored at −30 °C. The synthesis of callunene (**1**) was achieved in five steps. First, the 4-keto group of 4-oxoisoforone (**4**) was selectively protected using ethylene glycol in the presence of trimethyl orthoformate and *p*-toluenesulfonic acid to obtain ketal **5** (Figures S1 and S2). For the given reaction, flash chromatography was determined to be the superior purification method in comparison to the previously published vacuum distillation method.^18^ Then, ketal **5** was alkylated with the dianion of but-3-yn-2-ol, prepared by the addition of butyllithium to the solution of but-3-yn-2-ol in THF at −78 °C. The use of racemic but-3-yn-2-ol resulted in the formation of four diastereomers **6a**–**d**. When (2*S*)-but-3-yn-2-ol was used, only two diastereomers **6a** and **6b** were obtained. Diastereomers **6a**–**d** have very similar NMR spectra and are indistinguishable by standard NMR techniques (Figures S3 and S4). In the literature,^20,21^ separation of the two racemic diastereomers **6a**,**d** and **6b**,**c** by crystallization is described, with the comment that the stereochemical assignment of the diastereomers will be discussed in a forthcoming publication. However, we were unable to find the follow-up publication or repeat the separation. While the crystallization is complicated by the spontaneous hydrolysis of the acetal protecting group of alkyndiols **6** even at room temperature,^19^ we tried to achieve the crystallization using mixtures of solvents of different polarities.^20,21^ In all cases, only cocrystals of diastereomers were obtained (for the synthesis and characterization of the hydrolyzed product **6**, i.e., compound **S1**, see the Supporting Information). In the next step, the alkyndiols **6a**–**d** were transformed to allenic alcohols **7a**–**d** using LiAlH_4_ in anhydrous diethyl ether. It was observed that a temperature of 30 °C resulted in superior yields in comparison to 0 °C. However, the yields were relatively modest, primarily due to losses that occurred during the chromatography and the inherent instability of the acetal protecting group. Next, the stereogenic center at the 3′-position of alcohols **7a**–**d** was removed by oxidation of the 3′-hydroxy group to an oxo group by activated MnO_2_, giving two enantiomers of ketal-protected callunene **8a**,**b**. Final deprotection of the keto-group by mild acidic hydrolysis of **8a**,**b** with pyridinium *p*-toluenesulfonate (PPTS) in the mixture of water and acetone at 60 °C provided racemic callunene (**1a**,**b**) in 98% yield.

### Stereochemical Analysis

The absolute configuration of natural callunene (**1**) has not been previously described in the literature. Consequently, it remains unclear whether heather nectar contains a single enantiomer of callunene (**1a** or **1b**) or a racemic mixture (**1a,b**). Conducting biological assays on the individual enantiomers could, therefore, yield significant insights. Moreover, precise monitoring of the distribution of individual stereoisomers during the multistep synthesis of callunene (**1**) is crucial for the potential preparation of its individual enantiomers. Furthermore, the diastereomers of intermediates **6** and **7** show very similar NMR spectra. The absolute configuration of the 3′-stereogenic center of alkyndiols **6a**–**d** can be easily controlled using chiral (*2S*)-but-3-yn-2-ol in the synthesis. However, the nucleophilic addition of the lithium salt of but-3-yn-2-ol to the keto group of 4-protected 4-oxoisophorone **5** is not stereoselective. On the other hand, the formation of the allylic system by the reduction with LiAlH_4_ should be stereoselective due to its S_N_2′ mechanism.^22^ In the following steps, the absolute configuration of the allenic unit should remain stable. Therefore, monitoring the formation of specific diastereomers of intermediates **6** and **7** is essential. HPLC presents challenges due to the poor stability of the compounds. The use of acidic mobile phases during the HPLC run enhanced the separation; however, it resulted in partial loss of the protecting group. Although this method can still be used for the enrichment of the mixture with some of the stereoisomers, an accurate determination of the original stereoisomeric ratio remains problematic. To address this issue, we opted for ^1^H NMR methods using chiral solvating agents.

For our purpose, we have chosen deuterochloroform as a hydrogen-bonding noncompetitive solvent. As a solvating agent, we used three chiral reagents: (+)-Pirkle’s alcohol (**A**),^23^ (*S*_a_)-2-acetylamino-2′-[*N*′-3,5-bis(trifluoromethyl)phenyl]-ureido-1,1′-binaphthalene (**B**),^24^ and (*R*_a_)-2,2′-bis[*N*′-3,5-bis(trifluoromethyl)phenyl]ureido-1,1′-binaphthalene (**C**).^25^

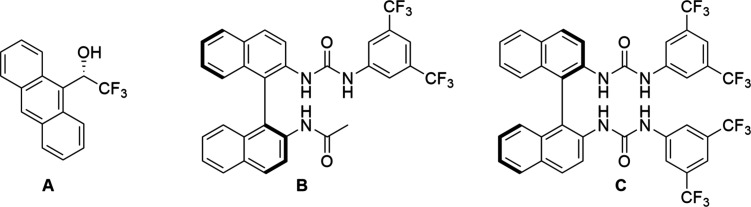



**Figure 1:**
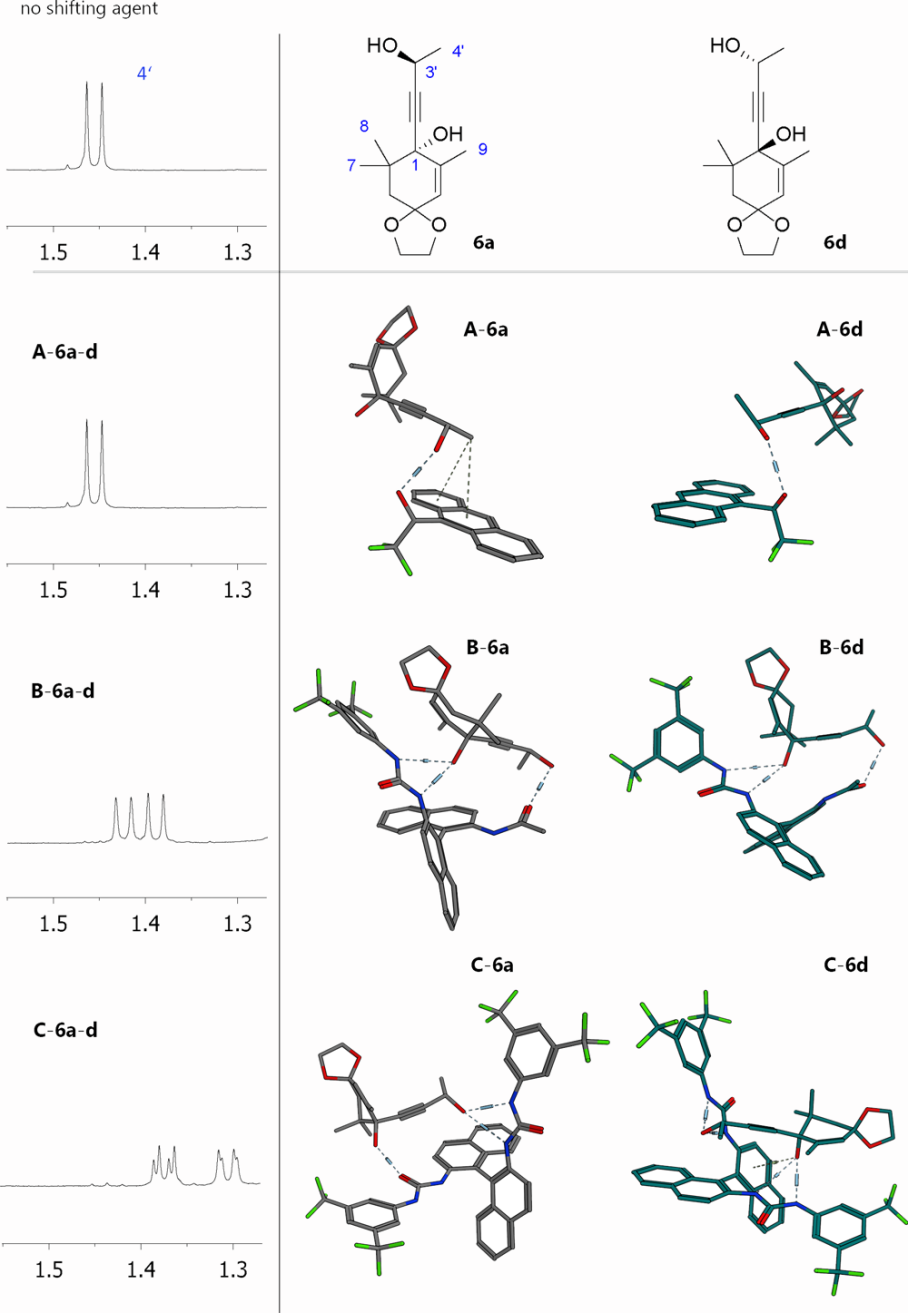
Chiral solvating agents **A**–**C** interacting with the mixture of **6a**–**d**. (Left) ^1^H NMR signal of methyl at position 4′ after addition of 3 equiv of respective shifting agent, 400 MHz, CDCl_3_, 25 °C. (Right) Calculated plausible structures of the complexes.

To better understand the formation of diastereomeric complexes with the shifting agents, molecular dynamics calculations were performed,^26^ and the results, along with the observed NMR signal splitting, are presented in Figure 1.

For diastereomeric mixture **6a**–**d**, a series of ^1^H NMR spectra were recorded at the starting concentration of 8 μM, adding 0.5, 1, 3, and 6 equiv of each shifting agent **A**, **B**, and **C**. At this concentration, reagent **A** did not cause any observable signal splitting of compound series **6a**–**d** (Figure 1, Figure S16). On the other hand, at a concentration of 0.1 M, the addition of 12 equiv of **A** can resolve signals of all four isomers of **6** at the signal of methyl 4′ (Figure S17). As confirmed by calculation, the complexes between diastereomers **6a**–**d** and reagent **A** are very weak, based only on the hydrogen bonding between hydroxy groups. In these complexes, the 4′-methyl signals of mixtures **6a**–**d** are resolved due to the different magnetic shielding caused by the spatial proximity of the anthracene aromatic system of **A.**

In the case of reagent **B**, at the 8 μM concentration of the diastereomeric mixture **6a**–**d**, we observed the separation of signals at positions 4′ and 7 after the addition of 0.5 equiv of the reagent. The addition of 3 equiv of **B** caused additional splitting of the signal at position 3 (Figure 1, Figures S18 and S19). However, we were able to observe only two sets of signals, although four stereoisomers **6a**–**d** were present. We assume separation of enantiomeric pair **6a**,**d** from **6b**,**c**, with the signals of diastereomers **6a**,**b** and **6c**,**d** occurring apparently at the same positions.

The addition of 3 equiv of the shifting reagent **C** at the 8 μM concentration of **6a**–**d** enabled the observation of resolved signals of all four stereoisomers **6a**–**d**. Again, we assume close proximity of the signals of diastereomeric pairs **6a**,**b** and **6c**,**d** and a more pronounced mutual separation of enantiomers. This premise is supported by the calculated structure of the complex for the (*R*,*R*)-enantiomer (**6d**). In the most represented complex, both OH groups of **6d** form hydrogen bonds O···H–N toward the NH group of the urea moiety of **C**. In the case of **6a**, the most represented complex shows H-bond between the 3′-OH group to urea oxygen via the hydrogen bond O—H···O=C. This makes a significant difference in the structures of complexes of enantiomers, as magnetic shielding in the **6a**-**C** complex is different from that in **6d**-**C**. On the other hand, the corresponding diastereomers differing at position 1 from the already assigned pair would not differ significantly from the already mentioned structures; thus, **6a**-**C** will be similar to **6b**-**C** and **6c**-**C** will be similar to **6d**-**C**, as the methyl in position 4′ is rather distant from the molecule of the shifting agent **C** (Figure 1, Figures S20–22).

**Figure 2:**
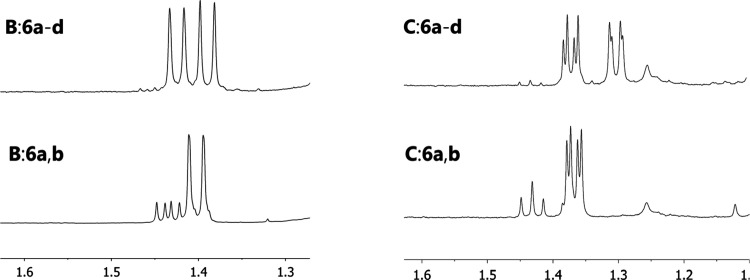
^1^H NMR signal of methyl at position 4′ after the addition of 3 equiv of the shifting agent for chiral solvating agents **B** and **C** interacting with the mixture of **6a**–**d** (top) and diastereomers **6a** and **6b** (bottom). ^1^H NMR, CDCl_3_, 400 MHz, 25 °C. The spectra of diastereomers **6a**,**b** contain signals of the ketal hydrolysis product **S1**.

Moreover, these spectra revealed an excess of one diastereomeric pair in the mixture. Integration of signals at position 3, after adding 6 equiv of **C** to **6a**-**d**, allowed us to estimate the ratio of **6a**,**b** to **6c**,**d** as approximately 2:3 (Figure S23). The unequal amount of the diastereomers in the mixture was visible in all split signals. The preferred signal separation of enantiomeric pairs over diastereomers by **B** or **C** was then undoubtedly confirmed by comparing the NMR spectra of the mixture of **6a**–**d** with that of the mixture containing only the two diastereomers, **6a** and **6b** (Figure 2, Figures S24 and S25). The opposite configuration of shifting agents **B** and **C** is reflected by the higher magnetic shielding observed for the opposite diastereomeric pairs. In the case of **B**, having a *S*_a_ configuration at the binaphthyl moiety, the **6a**,**b** pair of diastereomers is more shielded than **6c**,**d**. Solvating agent **C** with the *R*_a_ configuration showed the opposite behavior.

**Figure 3:**
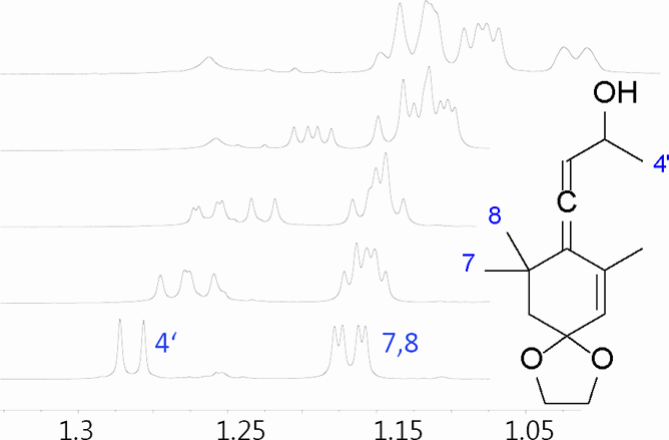
Separation of **7a**–**d** by the shifting agent **C**. Bottom to top: 0, 0.5, 1, 3, and 6 equiv of **C**. ^1^H NMR, CDCl_3_, 400 MHz, 25 °C.

Given the reliability and utility of the method employing chiral shifting agent **C** for monitoring the composition of the diastereomeric mixture **6a**–**d**, we extended its application to the separation of stereoisomers **7a**–**d**. These compounds possess a stereogenic center with central chirality and an axial allene moiety. The transformation of alkyndiols **6** to allenic alcohols **7** involves the loss of one OH group, which serves as a potential complexation site for the shifting agent. Consequently, we anticipated a weaker complexation ability of shifting agent **C** in proximity to the remaining 3′-OH group. The titration study confirmed this expectation, with the most pronounced signal splitting observed at the methyl hydrogens at position 4′ of **7a**–**d**. The separation of enantiomeric pairs **7a**,**d** and **7b**,**c** becomes evident even upon the addition of 0.5 equiv of the shifting agent **C**. At 1 equiv of **C**, one of the enantiomeric pairs resolves into diastereomers, and at 3 equiv the signals of this diastereomeric pair are fully resolved. However, the signals of the other enantiomeric pair are obscured due to overlap with the separating signals of the methyl groups at positions 7 and 8, rendering them unobservable (Figure 3, Figure S26).

**Figure 4:**
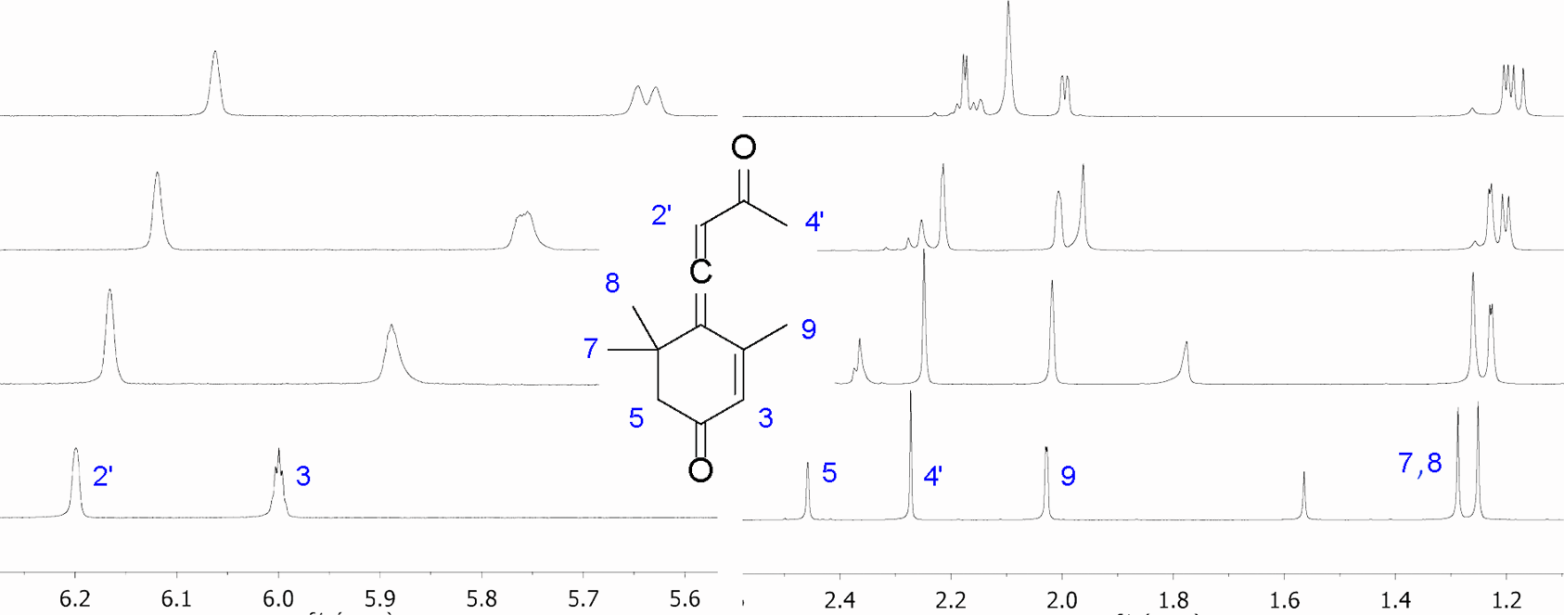
Titration of racemic callunene **1a**,**b** with **C**. Bottom to top: 0, 1, 3, and 6 equiv of **C**. ^1^H NMR, CDCl_3_, 400 MHz, 25 °C.

After the oxidation of allenic alcohols **7a**–**d** and the deprotection of **8a**,**b**, callunene (**1**) was obtained as a racemic mixture (**1a**,**b**). This racemic mixture was analyzed by titration with aliquots of chiral shifting agent **C** in deuterochloroform (Figure 4, Figure S27). Complexation with **C** involves two available keto groups, with our observations indicating a preferential interaction at the keto group on carbon 4. This interaction forms significantly stronger complexes compared with those observed with the hydroxy groups in stereoisomeric mixtures **6a**–**d** and **7a**–**d**. Although the urea-based binding site of **C** is rather sterically hindered and complexes other than those with 1:1 stoichiometry are unlikely, a Job plot analysis^27^ was performed to avoid any misinterpretation. Ten samples with different ratios of **1a**,**b** to **C** ranging from 0 to 6 equiv were measured, and the stoichiometry of the complex was confirmed to be 1:1 (Figure S31).

**Figure 5:**
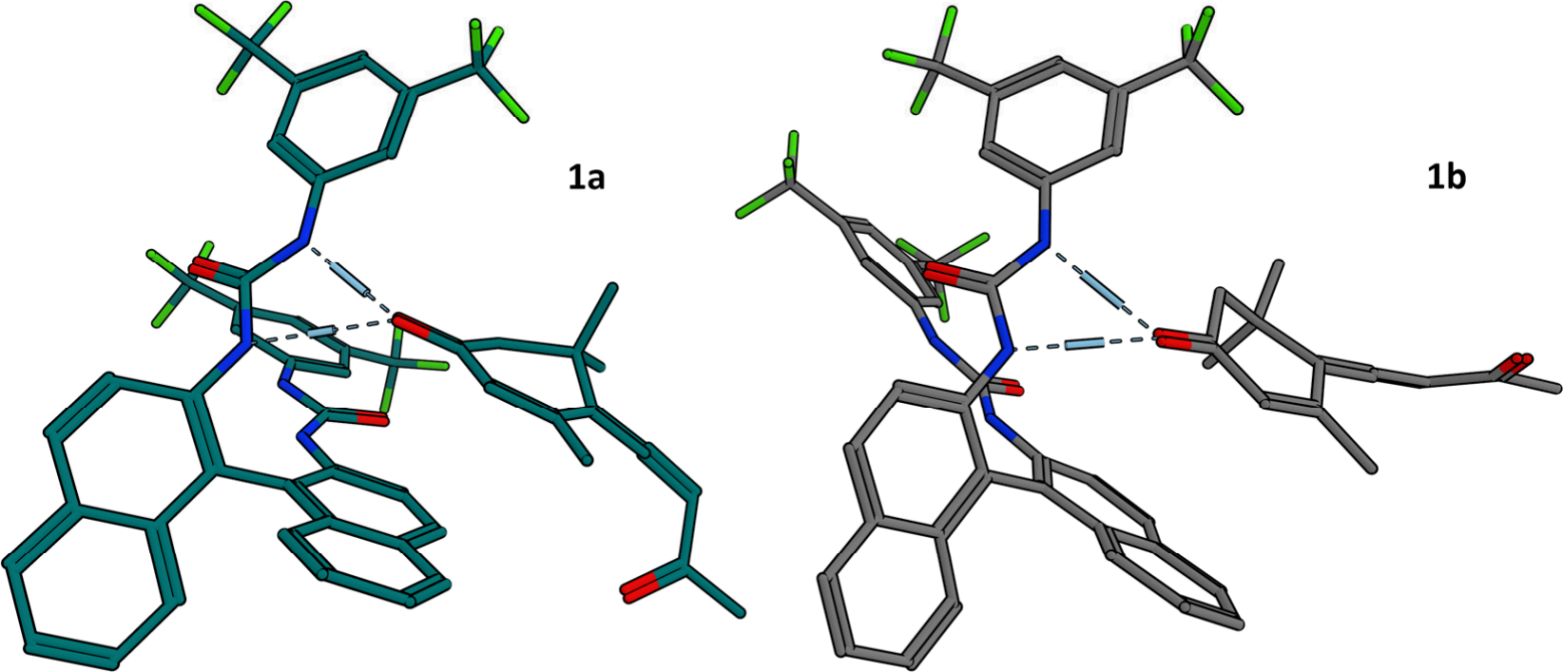
Calculated structures of complexes of (*S*_a_)-callunene (**1a**, green) and (*R*_a_)-callunene (**1b**, gray) with reagent **C**.

For racemic callunene (**1a**,**b**), the protons most affected by complexation are those at positions 3 and 5. These signals also exhibit the greatest splitting, reflecting differences in the magnetic shielding of these protons between the diastereomeric complexes. In the signals of the shifting agent, the most significant complexation-induced changes are observed at the protons attached to the CF_3_-substituted benzene rings and the proximal NH group (Figure S28). The plausible structure of the complex was determined by using computer-assisted molecular dynamics simulations (Figure 5, Figure S32). Although the complex itself is not very stable, and the position of callunene (**1a**,**b**) toward **C** is dynamic on average, the hydrogens in positions 3 and 5 are closer to reagent **C** for (*R*_a_)-callunene (**1b**) than for (*S*_a_)-callunene (**1a**). This results in stronger magnetic shielding of these protons for the *R*_a_-enantiomer compared to that for the *S*_a_-enantiomer, which is reflected in the lower field positioning of hydrogens at positions 3 and 5 of (*R*_a_)-callunene (**1b**).

**Figure 6:**
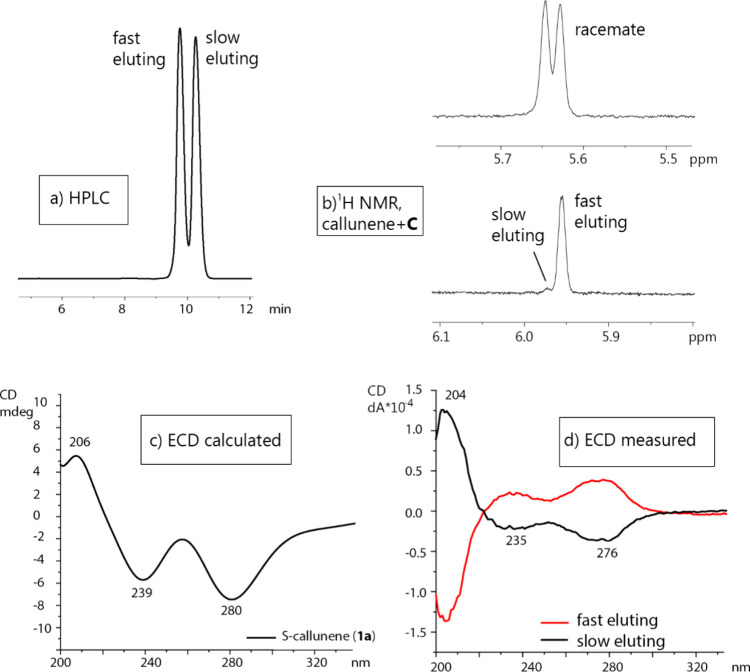
Absolute configuration assignment of callunene enantiomers (**1a**,**b**). (a) HPLC separation, (b) ^1^H NMR signal separation in the diastereomeric complex of **1a**,**b** (top) or fast eluting enantiomer (bottom) with shift reagent **C**, (c) calculated average ECD spectrum for *S*_a_-callunene (**1a**), and (d) measured ECD spectra of enantiomers **1a** and **1b**.

The assignment of the ^1^H NMR signals of the (*R*_a_)-callunene (**1b**) and (*S*_a_)-callunene (**1a**) complexes with **C** was confirmed by recording the spectra of one diastereomeric complex (Figure 6). For this purpose, the racemate was separated by chiral HPLC (Table S1, Figures S33 and S34). The best resolution was achieved with a Lux Amylose-3 column at 5 °C, using a mobile phase of heptane/propan-2-ol/diethylamine (95:5:0.1) containing 0.1% formic acid. Despite thorough mobile phase optimization, baseline resolution was not feasible. Therefore, both enantiomers for ECD analysis were collected from the effluent of the analytical column (Figure S33). The purities of the collected fast- and slow-eluting enantiomers were 84% and 85%, respectively (Figure S34). To the fast-eluting enantiomer, 6 equiv of **C** were added and the ^1^H NMR spectrum was recorded, showing the formation of the more shielded complex. The time-dependent density functional theory (TD-DFT) calculations were performed for (*S*_a_)-callunene (**1a**) and provided theoretical electronic circular dichroism (ECD) spectra as an average spectrum of the most probable conformers (Figure 6, Figures S35 and S36, Tables S2 and S3). Experimental ECD spectra were recorded for each of the HPLC-separated enantiomers. The comparison of the calculated and measured ECD spectra showed that the fast eluting peak was (*R*_a_)-callunene (**1b**) and the slow eluting one was (*S*_a_)-callunene (**1a**). The higher magnetic shielding of hydrogens at position 3 of (*R*_a_)-callunene (**1b**) in the complex with **C** observed in the ^1^H NMR spectrum is in accordance with the results obtained from the molecular dynamics simulations.

**Figure 7:**
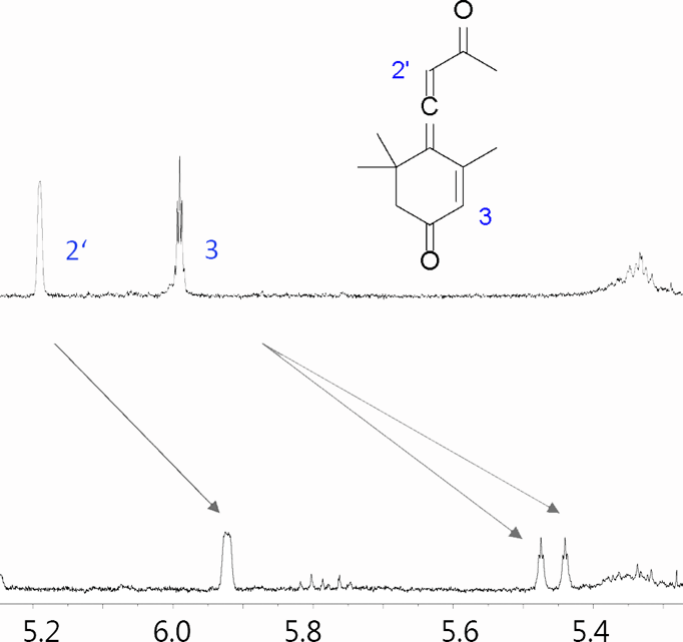
Enantioseparation of the signals of enantiomers of natural callunene (**1**) by reagent **C** (6 molar equiv). ^1^H NMR, CDCl_3_, 400 MHz, 25 °C.

The absolute configuration of natural callunene (**1**) was determined using callunene isolated from heather honey following a published procedure.^1^ The compound was purified from the crude extract by flash chromatography, followed by HPLC or preparative TLC. To ensure reliable results, two separate batches of commercial heather honey were processed. For ^1^H NMR studies, two samples of callunene (**1**) isolated from heather honey were used. 6 mol equiv of reagent **C** dissolved in deuterochloroform were added to each sample. In both cases, the signal for the hydrogen at position 3 was distinctly separated, unequivocally demonstrating the racemic nature of natural callunene (**1a**,**b**, Figure 7, Figures S29 and S30).

## Conclusions

The antiparasitic compound callunene (**1**) was synthesized, yielding quantities significantly greater than those obtainable from natural sources. A reliable method for the stereochemical analysis of callunene (**1**) and its synthetic intermediates **6** and **7** has been developed. The experimental findings from NMR experiments were further supported by the computational analysis of possible diastereomeric complexes of these compounds with (*R*_a_)-2,2′-bis[*N*′-3,5-bis(trifluoromethyl)phenyl]ureido-1,1′-binaphthalene (**C**). Using this approach, the naturally occurring callunene was found to be racemic. The chiral HPLC separation method has also been developed, enabling preparative separation of the enantiomers

The findings open new possibilities for the protection of indoor pollinators. As the callunene extracted from honey proved to be a racemate, it can easily be replaced with synthetically obtained callunene (**1**). The synthetic protocol can provide sufficient quantities for further studies on the mode of action and potential usage as a prophylactic additive to food of pollinators. The ability to obtain synthetic callunene (**1**) together with the possibility of observing its optical purity opens the way to possible derivatives with activity against a broader range of protozoal parasites.

## Experimental Section

### General Experimental Procedures

Optical rotations were measured at 25 °C, and [α]_D_ values are given in 10^–1^ deg cm^2^ g^–1^. The ECD/UV–vis spectra were recorded using the J-815 spectrometer (Jasco Corporation, Japan), which was purged with gaseous nitrogen (99.99%, Siad, Czech Republic). Spectra were recorded in a Suprasil quartz cuvette (Hellma, Germany) with a 1 mm path length in the 200–400 nm spectral region. The measurement was carried out at 20 °C with a scanning speed of 50 nm/min, a sensitivity of 100 mdeg, a 2 s response time, a bandwidth of 4 nm, a data pitch of 0.1 nm and in eight accumulations. For each of these eight accumulations, we calculated an average and then subtracted the spectrum of the mobile phase measured under identical experimental conditions. The absorbance was calculated from the photomultiplier HT voltage values using the Spectra analysis module of the Spectra Manager program, ver. 2.6.0.1 (Jasco Corporation, Japan). The initial geometries of (*S*_a_)-callunene were generated through a conformational search using the GFN-xTB method, implemented in the CREST (Conformer–Rotamer Ensemble Sampling Tool) program.^28^ All resulting conformers were subsequently optimized at the density functional theory (DFT) level in heptane, employing the iefpcm solvation model. Specifically, the hybrid functional mpw1pw91 and the cc-pvtz basis set were utilized for the optimizations. To obtain the ECD spectra, time-dependent DFT (TD-DFT) calculations were performed using the same functional and basis set.

The ^1^H and ^13^C spectra were recorded using Jeol 400 MHz (Jeol, Japan), Bruker Avance 400 MHz, and Bruker Avance III 600 MHz spectrometers (Bruker, Germany) at 25 °C in CDCl_3_. Chloroform-*d* was stored with molecular sieves. The ^1^H and ^13^C NMR spectra were referenced to the line of the solvent (δ/ppm; δH/δC: chloroform-d, 7.26/77.16). To assign all proton and carbon signals, a combination of 1D and 2D experiments (H,C-HSQC and H,C-HMBC) was used. Mass spectrometric data were obtained on an LTQ Orbitrap XL (Thermo Fisher Scientific) system at the Laboratory of Mass spectrometry, IOCB Prague. For thin-layer chromatography (TLC), silica gel plates Merck 60 F_254_ were used. Compounds were visualized on TLC plates by irradiation with UV light (254 nm) and/or with a solution of anisaldehyde (9 mL) in EtOH (230 mL), conc. H_2_SO_4_ (8 mL), and conc. AcOH (3 mL), followed by heating. Flash chromatography was performed using the Büchi Pure C-815 Flash system or Teledyne ISCO CombiFlash NextGen 300+ on CHROMABOND Flash empty cartridges filled with silica gel Silicycle-Siliaflash P 60 (particle size of 40–63 μm, pore diameter of 60 Å).

HPLC analyses were performed on an HPLC system ECS05 (ECOM spol. s r.o., Prague, Czech Republic) equipped with a solvent tray, a binary pump, a column oven, a diode array detector, an autosampler, and an integrated PC. The chromatographic instrument was controlled, and data were acquired using Clarity Chromatography Software (DataApex, Prague, Czech Republic). Chiral separation of racemic callunene was performed on a polysaccharide-based chiral stationary phase. From the five different amylose- and cellulose-based chiral stationary phases tested, the best result in terms of enantiomeric resolution was achieved on a Lux i-Amylose-3 column in a mobile phase composed of heptane/propan-2-ol (95:5, v/v) with formic acid (0.1%) and diethylamine (0.1%) as additives.

Reagents were purchased from commercial sources and used without further purification. Anhydrous solvents were dried by a PureSolv MD solvent purification system (Innovative Technology, Inc., USA).

All molecular modeling experiments were performed on a custom-made machine with Intel i9-12900K × 24 and NVIDIA RTX A5000 running Ubuntu 23.02. Molecular Operating Environment (MOE) 2022.2 and the Schrödinger suite (release 2023-2) were used as the main molecular modeling software packages.^26^ The single enantiomers and the chiral shifting agent molecules were built in MOE, and energy was minimized using the OPLS4 force field in Maestro. Molecular dynamics simulations were performed by using Desmond. All simulations were run in chloroform for 1000 ns, with a 2 fs time step, in the NPT ensemble with constant temperature (300 K) and pressure (1 atm). All other parameters were set using the default Desmond values. Images were created by using MOE.

### 7,9,9-Trimethyl-1,4-dioxaspiro[4.5]dec-6-en-8-one (**5**)

The synthesis of compound **5** was mostly based on the literature procedure^18^ with changes in purification of the final product. 4-Oxoisoforone **4** (10.00 g, 65.7 mmol), ethylene glycol (12.24 g, 197 mmol), *p*-toluenesulfonic acid monohydrate (454 mg, 2.64 mmol), and trimethyl-orthoformate (13.96 g, 132 mmol) were dissolved in dichloromethane (25 mL). The mixture was stirred for 4 h at 60 °C. The reaction progress was observed by TLC (hexane/ethyl acetate 5:1). Brine was added, and the mixture was extracted with diethyl ether. After being dried over magnesium sulfate, the organic phases were evaporated under reduced pressure. The product was purified by flash chromatography on silica using a gradient of EtOAc in hexane (0–30%). Product **5** was obtained as a yellow oil (11.08 g, 86% yield). ^1^H NMR (600 MHz, CDCl_3_) δ: 6.29 (d, *J* = 1.0 Hz, 1H), 4.02–3.99 (m, 4H), 2.05 (s, 2H, H5), 1.77 (d, *J* = 1.7 Hz, 3H), 1.17 (s, 6H). ^13^C NMR (151 MHz, CDCl_3_) δ: 204.2, 139.8, 135.6, 103.9, 64.7, 46.3, 42.1, 26.5, 16.3. HRMS (ESI) calcd for [C_11_H_17_O_3_]^+^: 197.11722. Found: 197.11732 [M + H]^+^.

### 4,4-(Ethylendioxy)-1-(3-hydroxybut-1-yn-1-yl)-2,6,6-trimethylcyclohex-2-en-1-ol (**6a**–**d**)

The synthesis of compounds **6a**–**d** was mostly based on the literature procedure,^19^ with changes in the purification of the final product. But-3-yn-2-ol (400 mg, 5.71 mmol) was dissolved in 8 mL of anhydrous THF in a flame-dried flask under an argon atmosphere. The mixture was cooled to −78 °C. After it was cooled, a solution of butyllithium (4.4 mL, 11 mmol, 2.5 M in hexane) was added dropwise on the flask wall within 30 min. The mixture was stirred at −78 °C for 30 min. In the separate flame-dried flask with molecular sieves (3 Å), ketal **5** (1.00 g, 5.10 mmol) was dissolved in anhydrous THF (2 mL). The solution of **5** was then added dropwise to the reaction mixture. The mixture spontaneously warmed to ambient temperature and was stirred overnight. The reaction progress was monitored using TLC (hexane/ethyl acetate 1:1). The remaining reagent was quenched with the addition of distilled water. The mixture was extracted with diethyl ether (3×). Combined organic layers were dried over magnesium sulfate and evaporated under reduced pressure. The crude product was purified by flash chromatography on silica, eluting with gradient of EtOAc in hexane (0–90%) to obtain a mixture of two racemic diastereomers **6a**–**d** (656 mg, 48% yield) as a yellowish oil. ^1^H NMR (600 MHz, CDCl_3_) δ: 5.35 (s, 1H), 4.55 (q, *J* = 6.5 Hz, 1H), 3.97–3.90 (m, 4H), 1.94–1.84 (m, 5H), 1.45 (d, *J* = 6.6 Hz, 3H), 1.13 (s, 3H), 1.09 (s, 3H). ^13^C NMR (151 MHz, CDCl_3_) δ 140.5, 123.6/123.5, 105.0, 84.2, 74.5, 64.5/64.3, 58.4, 43.8, 39.3, 25.6, 24.5/24.4, 22.7, 19.1. HRMS (ESI) calcd for [C_15_H_22_O_4_Na]^+^: 289.14103. Found: 289.14097 [M + Na]^+^.

### 4,4-(Ethylendioxy)-1-((*S*)-3-hydroxybut-1-yn-1-yl)-2,6,6-trimethylcyclohex-2-en-1-ol (**6a**,**b**)

A mixture of **6a** and **6b** was prepared as described for **6a**–**d** using (2*S*)-but-3-yn-2-ol (400 mg, 5.71 mmol) as a starting material. The mixture of diastereomers **6a**,**b** (712 mg, 53%) was obtained as a yellowish oil. [α]_D_: −17.1 (*c* = 0.291 g/100 mL). ^1^H NMR (600 MHz, CDCl_3_) δ 5.34 (s, 1H), 4.54 (q, *J* = 6.7 Hz, 1H), 3.97–3.90 (m, 4H), 1.94–1.84 (m, 5H), 1.44 (d, *J* = 6.6 Hz, 3H), 1.12 (s, 3H), 1.08 (s, 3H). ^13^C NMR (151 MHz, CDCl_3_) δ 140.6, 123.56/123.50, 105.0, 88.10/88.06, 84.2, 74.53/74.51, 64.4/64.3, 58.4, 43.8, 39.3, 25.6, 24.43/24.42, 22.7, 19.0. HRMS (ESI) calcd for [C_15_H_22_O_4_Na]^+^: 289.14103. Found: 289.14124 [M + Na]^+^.

Spontaneous hydrolysis of the ketal group of compound **6** occurred during storage, handling, and analysis. To confirm that the impurity occurring in the mixtures corresponds to this compound, we prepared and fully characterized the deprotected 4-hydroxy-4-(3-hydroxybut-1-yn-1-yl)-2,6,6-trimethylcyclohex-2-en-1-one (S1) as a diastereomeric mixture.

### 4,4-(Ethylenedioxy)-1-(3-hydroxybut-1-enyliden)-2,6,6-trimethylcyclohex-2-ene (**7a**–**d**)

The synthesis of compounds **7a**–**d** was mostly based on the literature procedure,^19^ with changes in reaction temperature and purification of the final product. The diastereomeric mixture **6a**–**d** (712 mg, 2.67 mmol) was evaporated with anhydrous acetonitrile (3×). The stirring bar was flame-dried and added to the flask under an argon atmosphere. Anhydrous diethyl ether (35 mL) was added, and the solution was cooled to 0 °C. A solution of lithium aluminum hydride (6.7 mL, 6.7 mmol, 1 M in THF) was added. The mixture was heated to 30 °C for 4 h. The reaction was monitored by TLC (hexane/ethyl acetate 1:1). After cooling, the reaction was quenched by addition of potassium sodium tartrate solution and brine. The product was extracted to diethyl ether (3×). Organic layers were combined, dried over magnesium sulfate, and evaporated. The product was purified by flash chromatography (silica, a gradient of EtOAc in hexane: 0–60%). The mixture **7a**–**d** (122 mg, 18%) was obtained as a yellow oil. ^1^H NMR (600 MHz, CDCl_3_) δ 5.64 (m, 1H), 5.46 (s, 1H), 4.36 (bs, 1H), 3.99–3.94 (m, 4H), 1.84 (s, 2H), 1.77 (t, *J* = 1.6 Hz, 3H), 1.31 (d, *J* = 6.6 Hz, 3H), 1.17–1.15 (4x s, 6H). ^13^C NMR (151 MHz, CDCl_3_) δ 200.6/200.5, 133.5/133.4, 124.1/124.0, 116.3/116.2, 105.6, 101.70/101.66, 66.44/66.41, 64.42/66.40, 46.2, 33.40/33.98, 30.0, 29.4, 23.64/23.60, 21.1. HRMS (EI) calcd for [C_15_H_22_O_3_]^+^: 250.1563. Found: 250.1566 [M]^+^.

### 4,4-(Ethylenedioxy)-1-(3-oxobut-1-enyliden)-2,6,6-trimethylcyclohex-2-ene (**8a**,**b**)

The synthesis of compounds **8a**,**b** was mostly based on the literature procedure,^19^ with changes the purification of the final product. Allenes **7a**–**d** (30 mg, 0.12 mmol) in hexane (5 mL) were added to a flame-dried flask under an argon atmosphere. After that, activated manganese(IV) oxide was added (208 mg, 2.39 mmol), and the mixture was stirred overnight at r.t. Next day, another portion of activated manganese(IV) oxide (107 mg, 1.23 mmol) was added, and the stirring continued for another three hours. The reaction was monitored by TLC (hexane/ethyl acetate 1:1). The solid content was filtered off, and the filtrate was evaporated and purified by flash chromatography (silica, a gradient of EtOAc in hexane: 0–60%) to obtain a racemic product **8a**,**b** (20 mg, 67%) as a yellowish oil. ^1^H NMR (600 MHz, CDCl_3_) δ 6.06 (s, 1H), 5.57 (s, 1H), 4.01–3.94 (m, 4H), 2.21 (s, 2H), 1.89 (s, 2H), 1.81 (d, *J* = 1.1 Hz, 3H), 1.56 (s, 2H), 1.27 (s, 3H), 1.22 (s, 3H). ^13^C NMR (151 MHz, CDCl_3_) δ: 213.6, 198.7, 130.9, 126.2, 117.0, 105.2, 102.5, 64.6, 46.2, 35.2, 29.5/29.4, 26.8, 21.0. HRMS (ESI) calcd for [C_15_H_20_O_3_Na]^+^: 271.13047. Found: 271.13024 [M + Na]^+^.

### 4-(3-Oxobut-1-enylidene)-3,5,5-trimethylcyclohex-2-en-1-one (Callunene, **1a**,**b**)

Pyridinium *p*-toluenesulfonate (PPTS, 37 mg, 0.15 mmol) was added to a solution of allenic ketone **8a**,**b** (121 mg, 0.49 mmol) in a mixture of acetone (6 mL) and distilled water (0.9 mL). The mixture was heated to 60 °C for 1 h. After evaporation, the crude product was purified by flash chromatography (silica, 10–30% EtOAc in hexane) to give callunene (**1a**,**b**, 87 mg, 87%) as a colorless amorphous solid. ^1^H NMR (400 MHz, CDCl_3_) δ 6.19 (s, 1H, H2′), 5.99 (m, 1H, H3), 2.45 (s, 2H, H5), 2.26 (s, 3H, C4′), 2.02 (d, *J* = 0.7 Hz, 3H, H9), 1.28 (s, 3H, H7), 1.24 (s, 3H, H8). ^13^C NMR (101 MHz, CDCl_3_) δ: 214.3 (C1′), 197.5 (C3′), 197.1 (C4), 148.5 (C2), 127.3 (C3), 117.1 (C1), 102.6 (C2′), 50.9 (C5), 37.1 (C6), 28.8 (C7), 28.7 (C8), 27.3 (C4′), 21.6 (C9). HRMS (EI): calcd for [C_13_H_16_O_2_]^+^: 204.1145. Found: 204.1140 [M]^+^.

### Isolation of Callunene from Heather Honey

Natural callunene was isolated from heather honey (Raw *Calluna* honey, Goodie, s.r.o., Czech Republic, country of origin: Spain) in analogy with a published procedure.^1^ One batch of honey was separated into portions of approximately 100 g, and these portions were extracted separately. Approximately 100 g of honey was dissolved in water (440 mL), saturated brine (130 mL) was added, and the solution was extracted with hexane (2× 166 mL). Then, the combined organic phases were washed with saturated brine (130 mL), dried over sodium sulfate, and evaporated. The crude extract was separated by flash chromatography (4 g of silica, dry loading on Celite, gradient: 0–25% EtOAc in cyclohexane). Callunene was further purified by either HPLC or preparative TLC. HPLC purification: Büchi Pure C-850 FlashPrep system with a column packed with 5 μm normal phase (ProntoSIL 60–5-Si 150 × 20 mm, BISCHOFF Chromatography); flow rate: 15 mL/min; gradient of EtOAc in hexane: 0% for 5 min, 0–15% for 30 min, 15–20% for 5 min. TLC purification: cyclohexane/EtOAc 2:1. Two batches of honey were used for the isolation. Batch #1: 320 g of honey provided 321 mg of crude extract; after flash chromatography and HPLC purification, 2.7 mg of callunene was obtained. Batch #2: 400 g of honey provided 187 mg of crude extract; after flash chromatography and preparative TLC purification, 5.0 mg of callunene was obtained.

## Supporting Information Available

Experimental data on the synthesis of **S1**, copies of NMR spectra of compounds **1**–**8** and **S1**, NMR spectra of titration experiments with NMR shift reagents, HPLC enantioseparation, and ECD spectra calculations (PDF)

## References

[ref1] Koch H.; Woodward J.; Langat M. K.; Brown M. J. F.; Stevenson P. C. Flagellum Removal by a Nectar Metabolite Inhibits Infectivity of a Bumblebee Parasite. Curr. Biol. 2019, 29 (20), 3494–3500. 10.1016/j.cub.2019.08.037. 31607528

[ref2] Tan S. T.; Wilkins A. L.; Holland P. T.; McGhie T. K. Extractives from New Zealand Unifloral Honeys. 2. Degraded Carotenoids and Other Substances from Heather Honey. J. Agr. Food Chem. 1989, 37 (5), 1217–1221. 10.1021/jf00089a004.

[ref3] Sefton M. A.; Francis I. L.; Williams P. J. Free and Bound Volatile Secondary Metabolites of Vitis Vinifera Grape Cv. Sauvignon Blanc. J. Food Sci. 1994, 59 (1), 142–147. 10.1111/j.1365-2621.1994.tb06919.x.

[ref4] Yourth C. P.; Brown M. J. F.; Schmid-Hempel P. Effects of Natal and Novel Crithidia Bombi (Trypanosomatidae) Infections on Bombus Terrestris Hosts. Insect. Soc. 2008, 55 (1), 86–90. 10.1007/s00040-007-0974-1.

[ref5] Heathlands: Patterns and Processes in a Changing Environment; Aerts R., Heil G. W., Eds.; Kluwer Academic Publishers, 1993.

[ref6] Fagúndez J. Heathlands Confronting Global Change: Drivers of Biodiversity Loss from Past to Future Scenarios. Ann. Bot. 2013, 111 (2), 151–172. 10.1093/aob/mcs257. 23223202 PMC3555525

[ref7] Kang B.-K.; Kim M.-J.; Kim K.-B.-W.-R.; Ahn D.-H. In Vivo and in Vitro Inhibitory Activity of an Ethanolic Extract of Sargassum Fulvellum and Its Component Grasshopper Ketone on Atopic Dermatitis. Int. Immunopharmacol. 2016, 40, 176–183. 10.1016/j.intimp.2016.07.015. 27608302

[ref8] Kim M.-J.; Jeong S.-M.; Kang B.-K.; Kim K.-B.-W.-R.; Ahn D.-H. Anti-Inflammatory Effects of Grasshopper Ketone from Sargassum Fulvellum Ethanol Extract on Lipopolysaccharide-Induced Inflammatory Responses in RAW 264.7 Cells. J. Microbiol. Biotechn. 2019, 29 (5), 820–826. 10.4014/jmb.1901.01027. 30982318

[ref9] Yue X.-D.; Qu G.-W.; Li B.-F.; Xue C.-H.; Li G.-S.; Dai S.-J. Two New C13 -Norisoprenoids from Solanum Lyratum. J. Asian Nat. Prod. Res. 2012, 14 (5), 486–490. 10.1080/10286020.2012.678331. 22530676

[ref10] Ceramella J.; Iacopetta D.; Franchini A.; De Luca M.; Saturnino C.; Andreu I.; Sinicropi M. S.; Catalano A. A Look at the Importance of Chirality in Drug Activity: Some Significative Examples. Appl. Sci. 2022, 12 (21), 10909 10.3390/app122110909.

[ref11] Waldeck B. Biological Significance of the Enantiomeric Purity of Drugs. Chirality 1993, 5 (5), 350–355. 10.1002/chir.530050514. 8398592

[ref12] Xie S.; Yuan L. Recent Progress of Chiral Stationary Phases for Separation of Enantiomers in Gas Chromatography. J. of Sep. Sci. 2017, 40 (1), 124–137. 10.1002/jssc.201600808. 27570052

[ref13] Chankvetadze B. Recent Developments on Polysaccharide-Based Chiral Stationary Phases for Liquid-Phase Separation of Enantiomers. J. Chromatogr. A 2012, 1269, 26–51. 10.1016/j.chroma.2012.10.033. 23141986

[ref14] Papp L. A.; Szabó Z. I.; Hancu G.; Farczádi L.; Mircia E. Comprehensive Review on Chiral Stationary Phases in Single-Column Simultaneous Chiral-Achiral HPLC Separation Methods. Molecules 2024, 29 (6), 1346 10.3390/molecules29061346. 38542982 PMC10975973

[ref15] Wenzel T. J. Discrimination of chiral compounds using NMR spectroscopy; Wiley: Hoboken, NJ, 2007.

[ref16] Wenzel T. J. Enantiomeric Purity Studied Using NMR. In Encyclopedia of Spectroscopy and Spectrometry; Lindon J. C., Tranter G. E., Koppenaal D. W., Eds.; Elsevier, 2017; pp 490–502.

[ref17] Balzano F.; Uccello-Barretta G.; Aiello F. Chiral Analysis by NMR Spectroscopy: Chiral Solvating Agents. In Chiral Analysis, 2nd ed.; Elsevier, 2018; pp 367–427.

[ref18] Weyerstahl P.; Meisel T.; Mewes K.; Negahdari S. Struktur Und Geruch, XIII. Synthese Und Olfaktorische Eigenschaften von Megastigmatrienon-Analoga. Liebigs Ann. Chem. 1991, 1991 (1), 19–25. 10.1002/jlac.199119910104.

[ref19] Abrams S. R.; Milborrow B. V. Synthesis and Biological Activity of Allenic Analogues of Abscisic Acid. Phytochemistry 1991, 30 (10), 3189–3195. 10.1016/0031-9422(91)83174-J.

[ref20] Demole E.; Enggist P. Novel Synthesis of 3,5,5-Trimethyl-4-(2-butenylidene)-cyclohex-2-en-1-one, a Major Constituent of Burley Tobacco Flavour. Helv. Chim. Acta 1974, 57 (7), 2087–2091. 10.1002/hlca.19740570722.

[ref21] Weyerstahl P.; Meisel T. Structure-Odor Correlation, XIX. Synthesis and Olfactory Properties of Various Racemic Theaspirones, Ketoedulans and Edulans. Liebigs Ann. Chem. 1994, 1994 (4), 415–427. 10.1002/jlac.199419940415.

[ref22] Krause N.; Hoffmann-Röder A. Synthesis of Allenes with Organometallic Reagents. Tetrahedron 2004, 60 (51), 11671–11694. 10.1016/j.tet.2004.09.094.

[ref23] Pirkle W. H.; Sikkenga D. L.; Pavlin M. S. Nuclear Magnetic Resonance Determination of Enantiomeric Composition and Absolute Configuration of.gamma.-Lactones Using Chiral 2,2,2-Trifluoro-1-(9-Anthryl)ethanol. J. Org. Chem. 1977, 42 (2), 384–387. 10.1021/jo00422a061.

[ref24] Holakovský R.; März M.; Cibulka R. Urea Derivatives Based on a 1,1′-Binaphthalene Skeleton as Chiral Solvating Agents for Sulfoxides. Tetrahedron Asymmetr. 2015, 26 (23), 1328–1334. 10.1016/j.tetasy.2015.10.011.

[ref25] Cuřínová P.; Dračínský M.; Jakubec M.; Tlustý M.; Janků K.; Izák P.; Holakovský R. Enantioselective Complexation of 1-phenylethanol with Chiral Compounds Bearing Urea Moiety. Chirality 2018, 30 (6), 798–806. 10.1002/chir.22855. 29578615

[ref26] aMolecular Operating Environment, ver. 2022.2; Chemical Computing Group, 2022.

[ref27] Hirose K. A Practical Guide for the Determination of Binding Constants. J. Incl. Phenom. Macro. 2001, 39 (3), 193–209. 10.1023/A:1011117412693.

[ref28] Pracht P.; Grimme S.; Bannwarth C.; Bohle F.; Ehlert S.; Feldmann G.; Gorges J.; Müller M.; Neudecker T.; Plett C.; Spicher S.; Steinbach P.; Wesołowski P. A.; Zeller F. J. Chem. Phys. 2024, 160, 114110 10.1063/5.0197592. 38511658

