# Supplementary material for: Stereoanalysis of the Antiparasitic Natural Product Callunene and Its Synthetic Intermediates
Source: J Nat Prod. 2025 Mar 3;88(3):723–31. doi: 10.1021/acs.jnatprod.4c01424 (PMC11959598; doi:10.1021/acs.jnatprod.4c01424)
Supplement: Supplementary file 1 — np4c01424_si_001.pdf [file np4c01424_si_001.pdf]

Supporting information for

Stereo-analysis of the antiparasitic natural product callunene and its synthetic intermediates

Jiří Ferenczei, [a,b] Vilém Blahout, [a] Hana Dvořáková, [b] Andrea Brancale, [a] Petra Cuřínová, [a,*] Magdaléna Labíková, [a] Michal Kohout, [a] Vladimír Setnička, [c] and Pavla Perlíková [a,d,*]

[a] *Department of Organic Chemistry, Faculty of Chemical Technology, University of Chemistry and Technology Prague, Technická 5, Prague 6, 16628, Czech Republic*

[b] *Laboratory of Nuclear Magnetic Resonance Spectroscopy, University of Chemistry and Technology Prague, Technická 5, Prague 6, 16628, Czech Republic*

[c] *Department of Analytical Chemistry, Faculty of Chemical Engineering, University of Chemistry and Technology Prague, Technická 5, Prague 6, 16628, Czech Republic*

[d] *Institute of Organic Chemistry and Biochemistry, Czech Academy of Sciences, Flemingovo nám. 2, Prague 6, 16000, Czech Republic*

Contents

Synthesis of compound **S1** 2

NMR spectra 3

Calculated complex structures 20

HPLC separation 21

DFT geometry optimization 22

Synthesis of compound **S1**

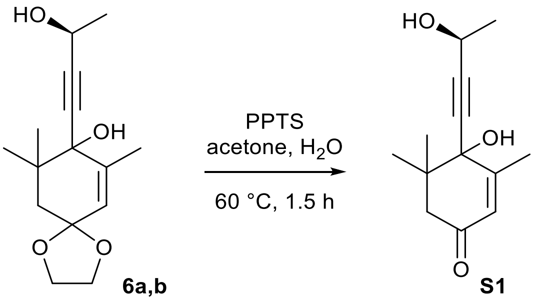

4-Hydroxy-4-(3-hydroxybut-1-yn-1-yl)-2,6,6-trimethylcyclohex-2-en-1-one (S1)

The diastereomeric mixture **6a,b** (30 mg, 0.11 μmol) was dissolved in the mixture of distilled water (450 μL) and acetone (3 mL). Pyridinium *p*-toluenesulfonate (PPTS, 9.0 mg, 36 μmol) was added and the mixture was heated to 60 °C for 90 minutes. TLC (hexane:ethyl acetate 1:1) . The product was directly purified by flash chromatography (silica, eluted by gradient EtOAc in hexane (0-60%). After purification, a mixture of diastereomers **S1** was obtained as a yellowish oil (15 mg, 59%).

^{1}H NMR (600 MHz, $CDCl_3$) δ: 5.85 (s, 1H, H3), 4.60 (q, J = 6.2 Hz, 1H, H3′), 2.52-2.38 (d, J = 17 Hz, 2H, H5), 2.11 (s, 3H, H9), 1.48 (d, J = 6.5 Hz, 3H, H4′), 1.21 (s, 3H, H7), 1.10 (s, 3H, H8).

^{13}C NMR (151 MHz, $CDCl_3$) δ: 198.1 (C4), 159.5 (C2, from HMBC), 126.4 (C3), 89.4 (C2′), 82.8 (C1′), 74.6 (C1), 58.5 (C3′), 50.1 (C5), 41.9 (C6), 25.2 (C7,8), 24.5 (C4′), 19.7 (C9).

HRMS (ESI): calc for $[C_{13}H_{18}O_3Na]^+$: 245.11505; found: 245.11502 $[M+Na]^+$

NMR spectra

Spectral identification of compounds **5-8** and callunene (**1a,b**).

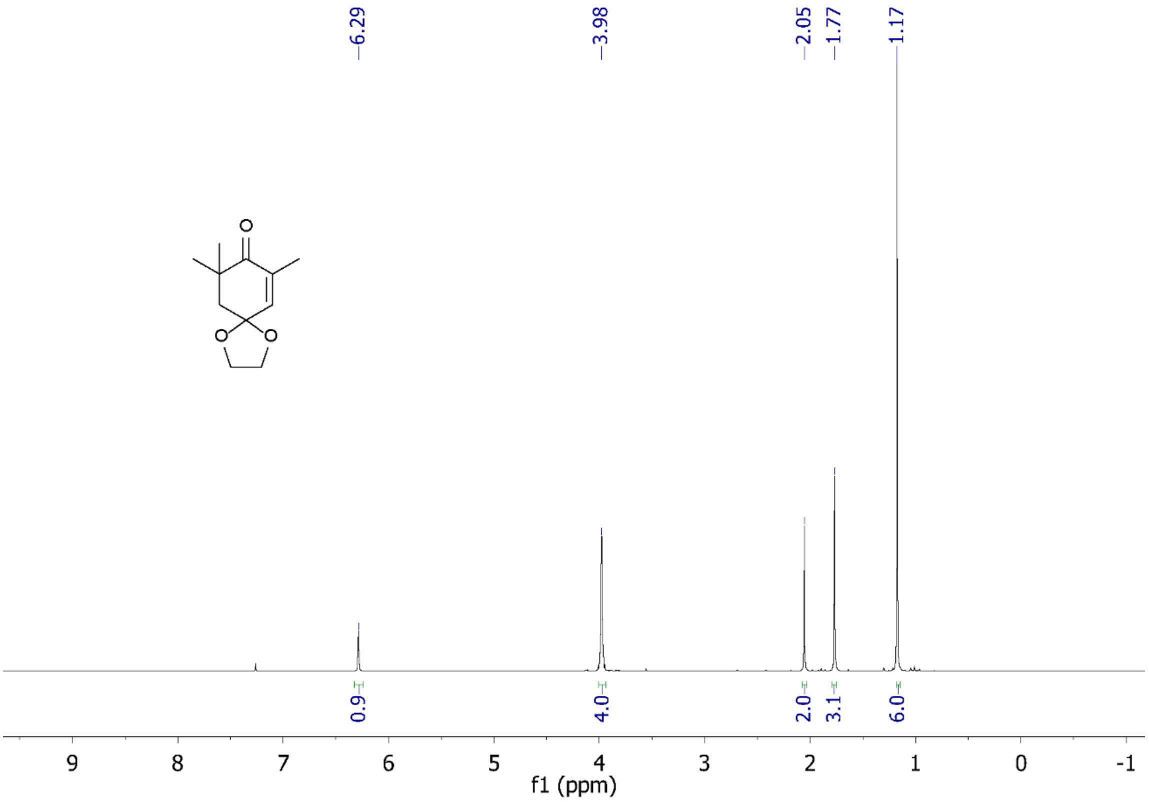

Figure S1. ^{1}H NMR spectrum of compound **5** (600 MHz, 25°C, $CDCl_3$).

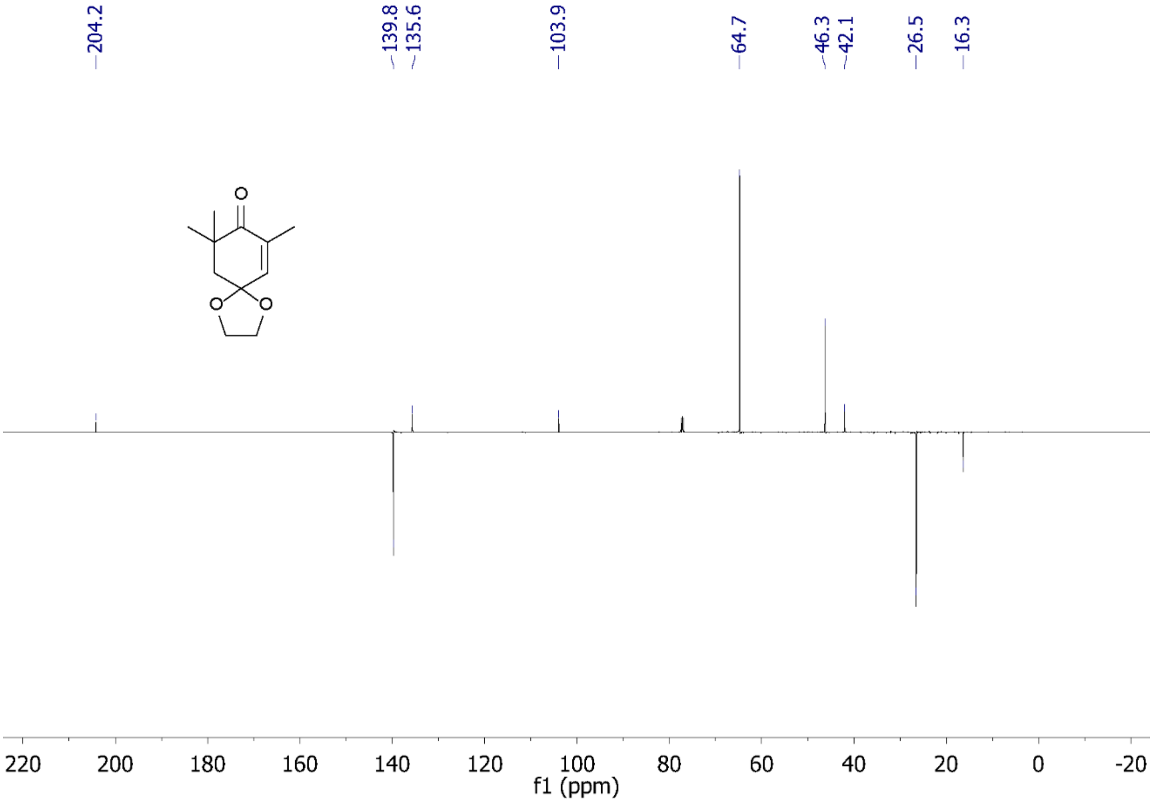

Figure S2. ^{13}C APT NMR spectrum of compound **5** (151 MHz, 25°C, $CDCl_3$).

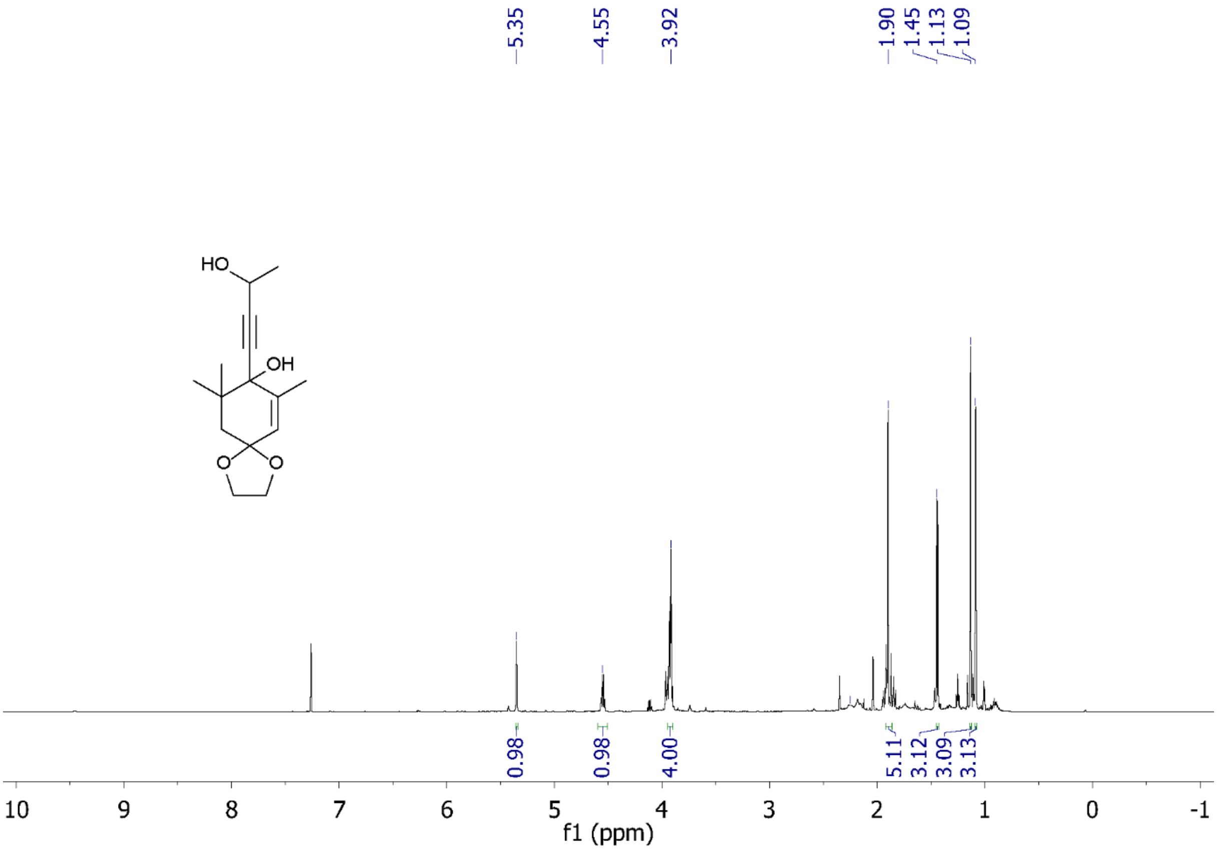

Figure S3. ^{1}H NMR spectrum of compounds **6a-d** (600 MHz, 25°C, $CDCl_3$).

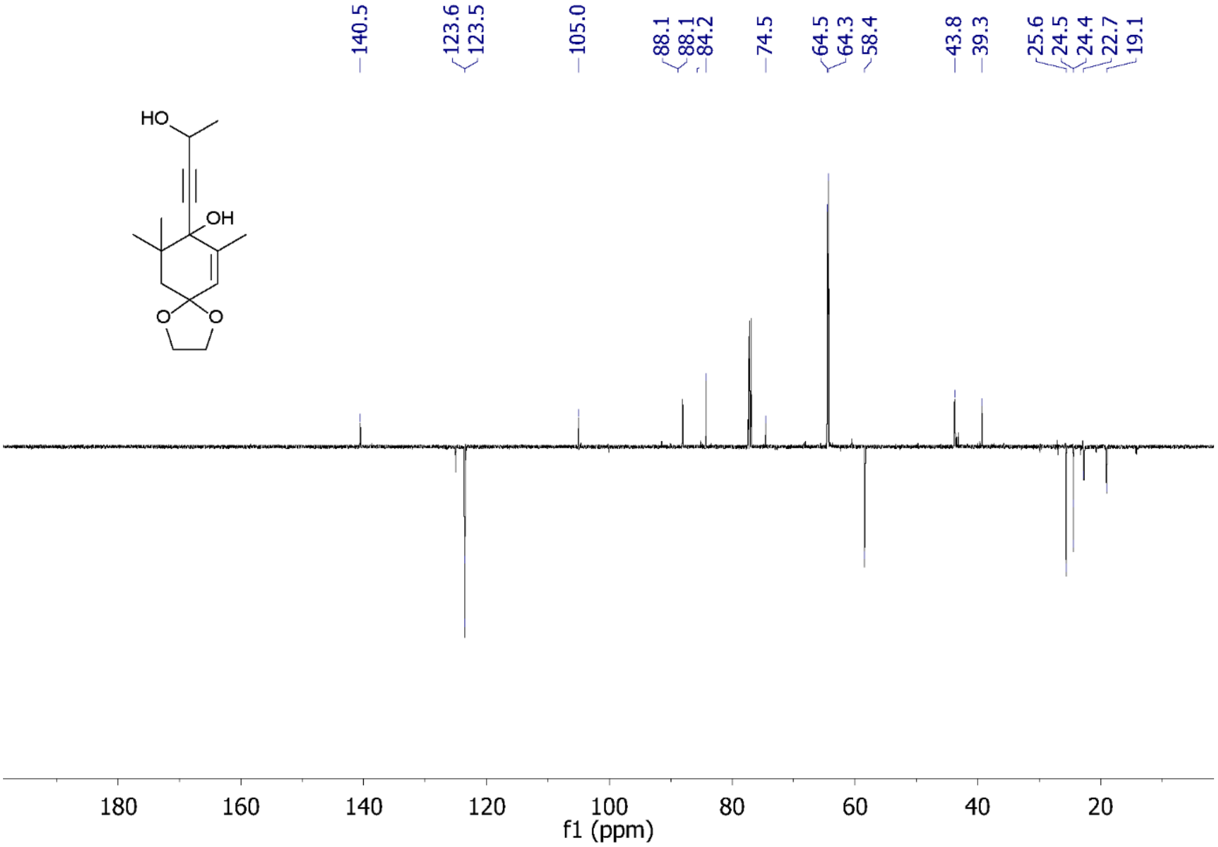

Figure S4. ^{13}C APT NMR spectrum of compounds **6a-d** (151 MHz, 25°C, $CDCl_3$).

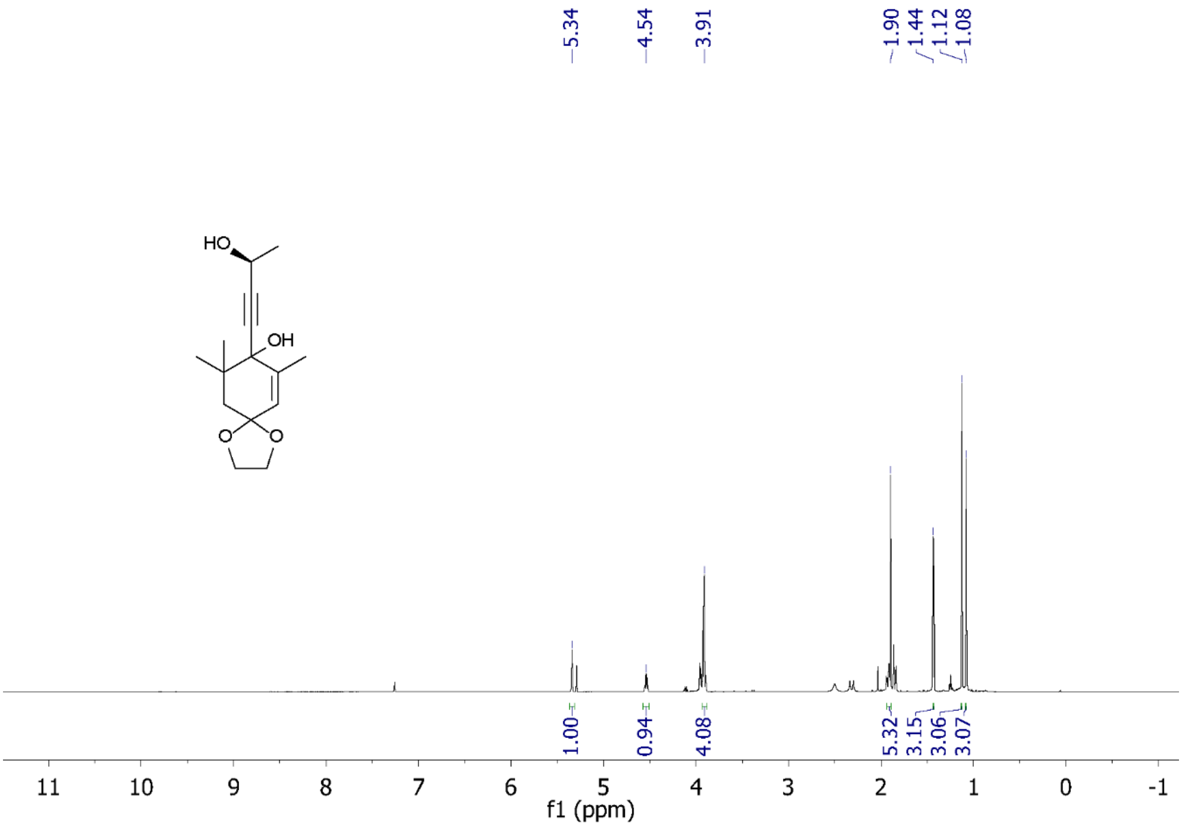

Figure S5. ^{1}H NMR spectrum of compounds **6a,b** (600 MHz, 25°C, $CDCl_3$).

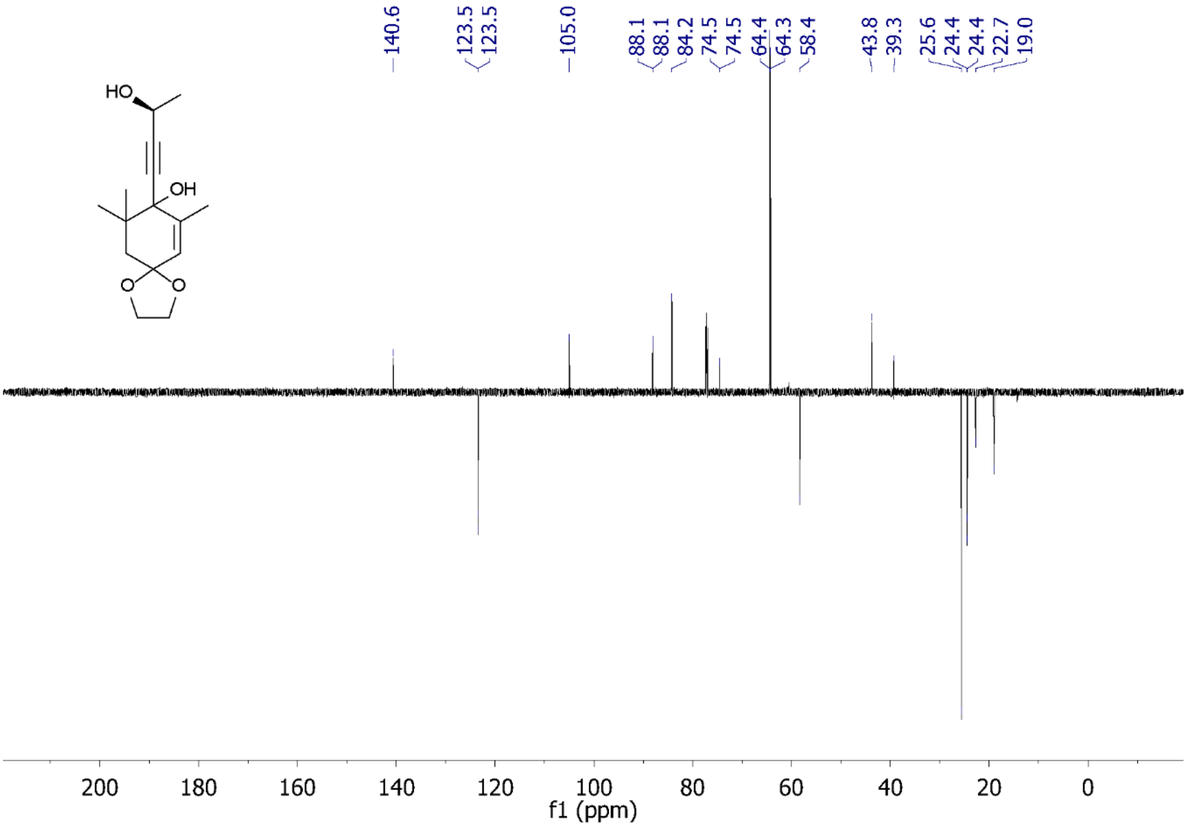

Figure S6. ^{13}C APT NMR spectrum of compounds **6a,b** (151 MHz, 25°C, $CDCl_3$).

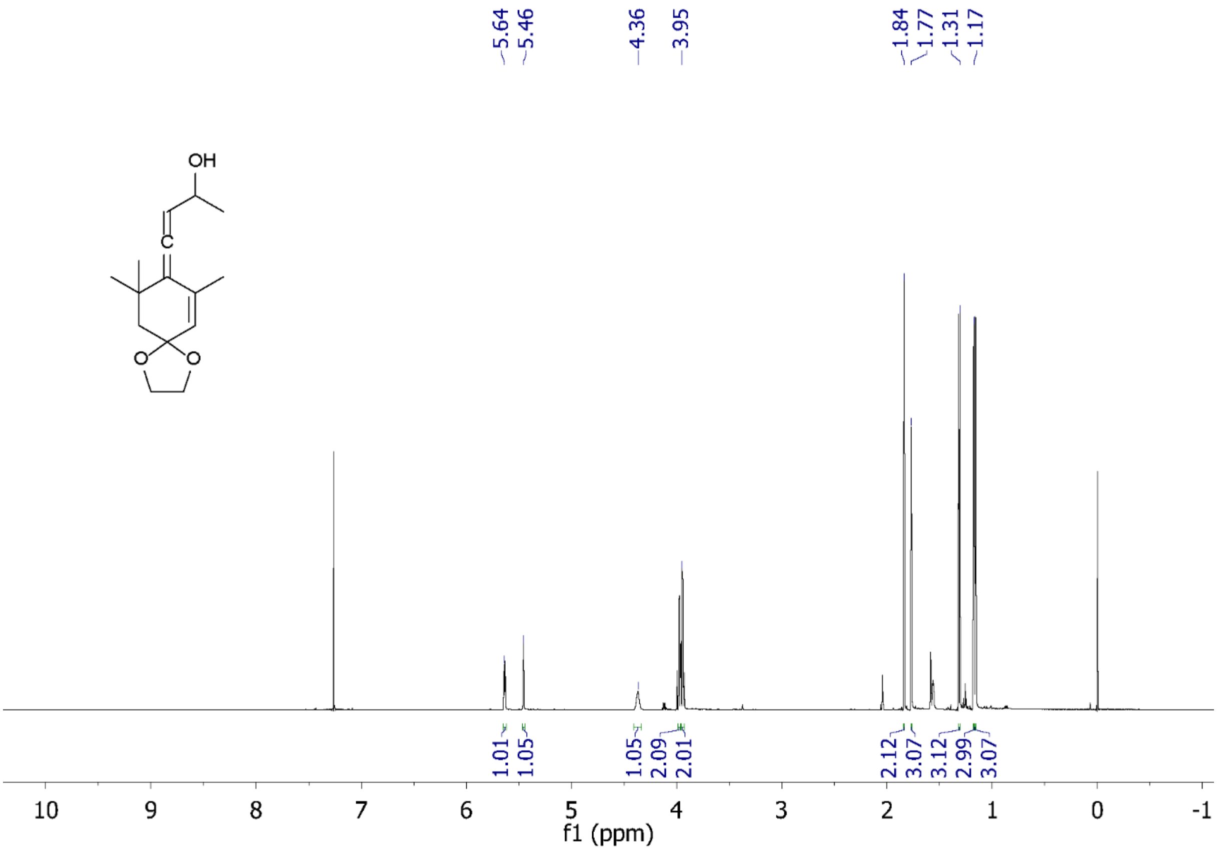

Figure S7. ^{1}H NMR spectrum of compounds **7a-d** (600 MHz, 25°C, $CDCl_3$).

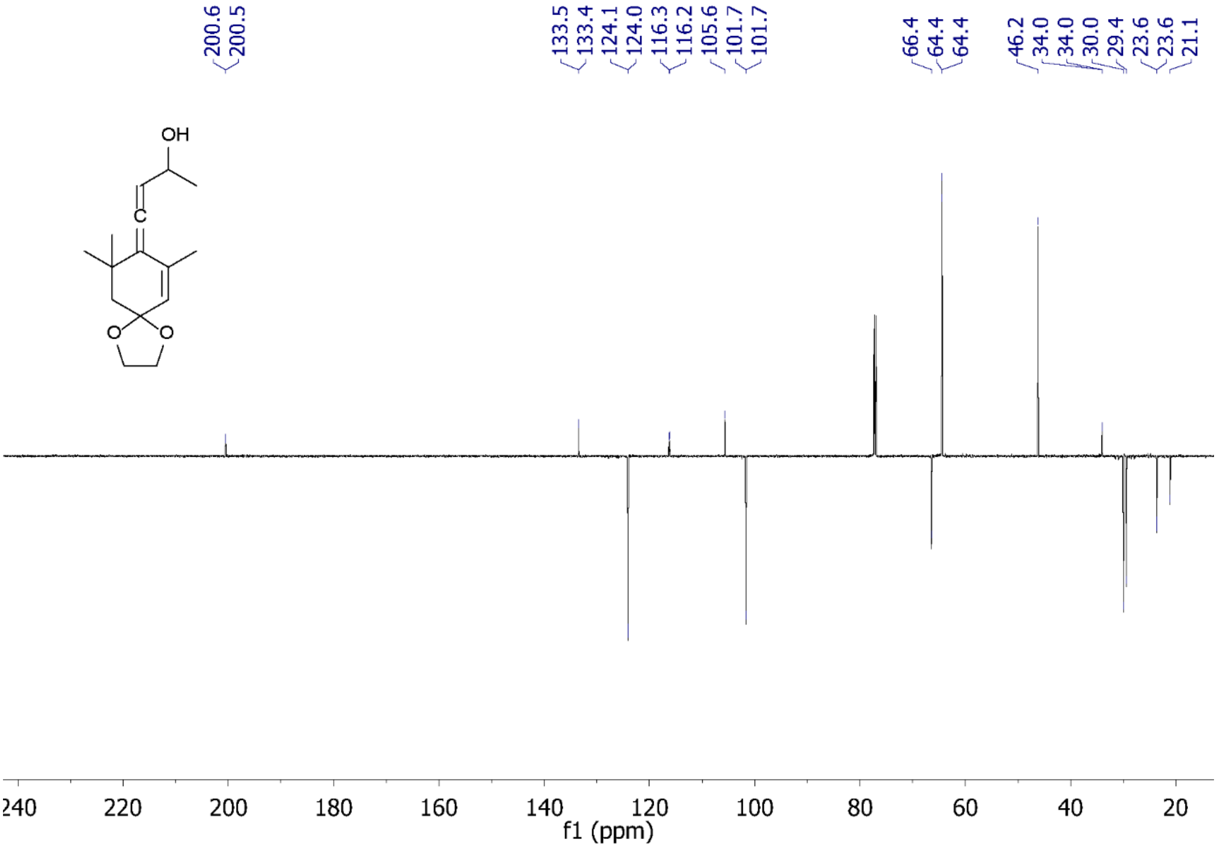

Figure S8. ^{13}C APT NMR spectrum of compounds **7a-d** (151 MHz, 25°C, $CDCl_3$).

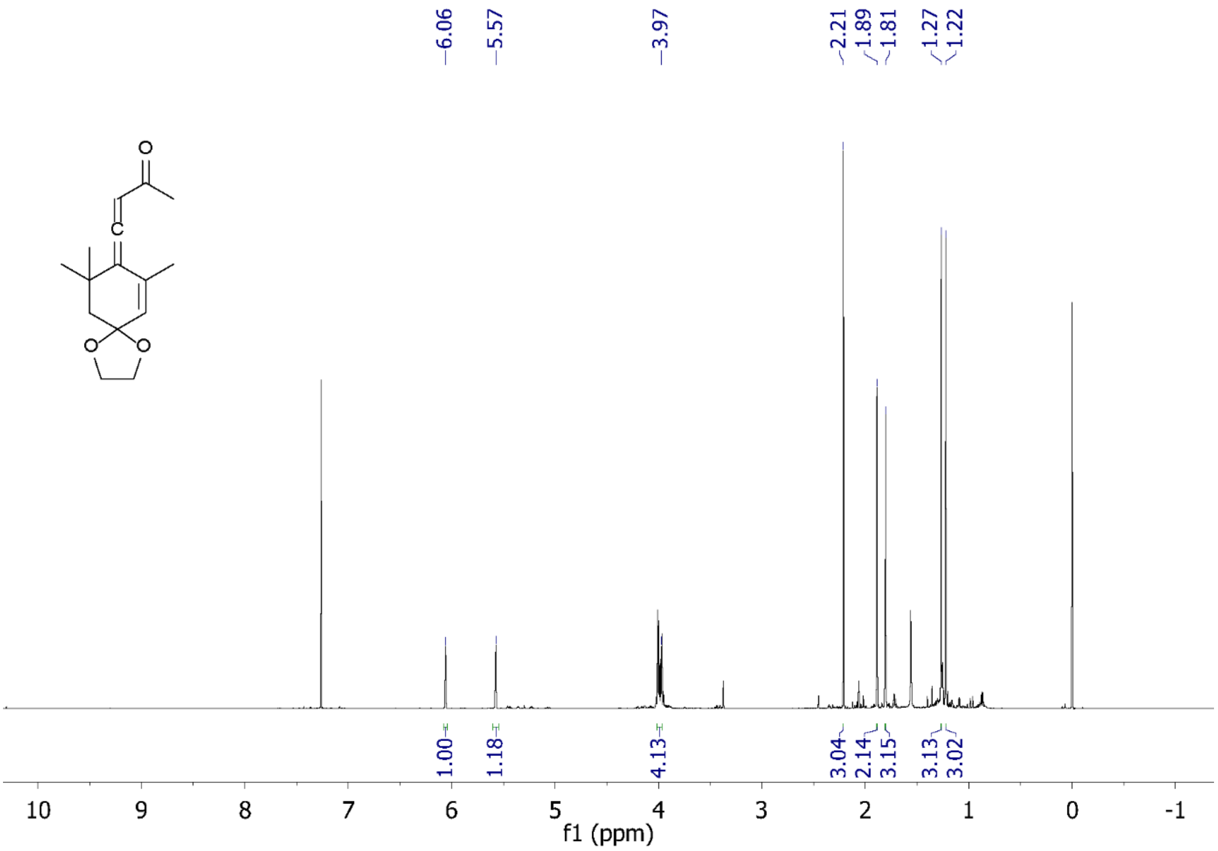

Figure S9. ^{1}H NMR spectrum of compounds **8a,b** (600 MHz, 25°C, $CDCl_3$).

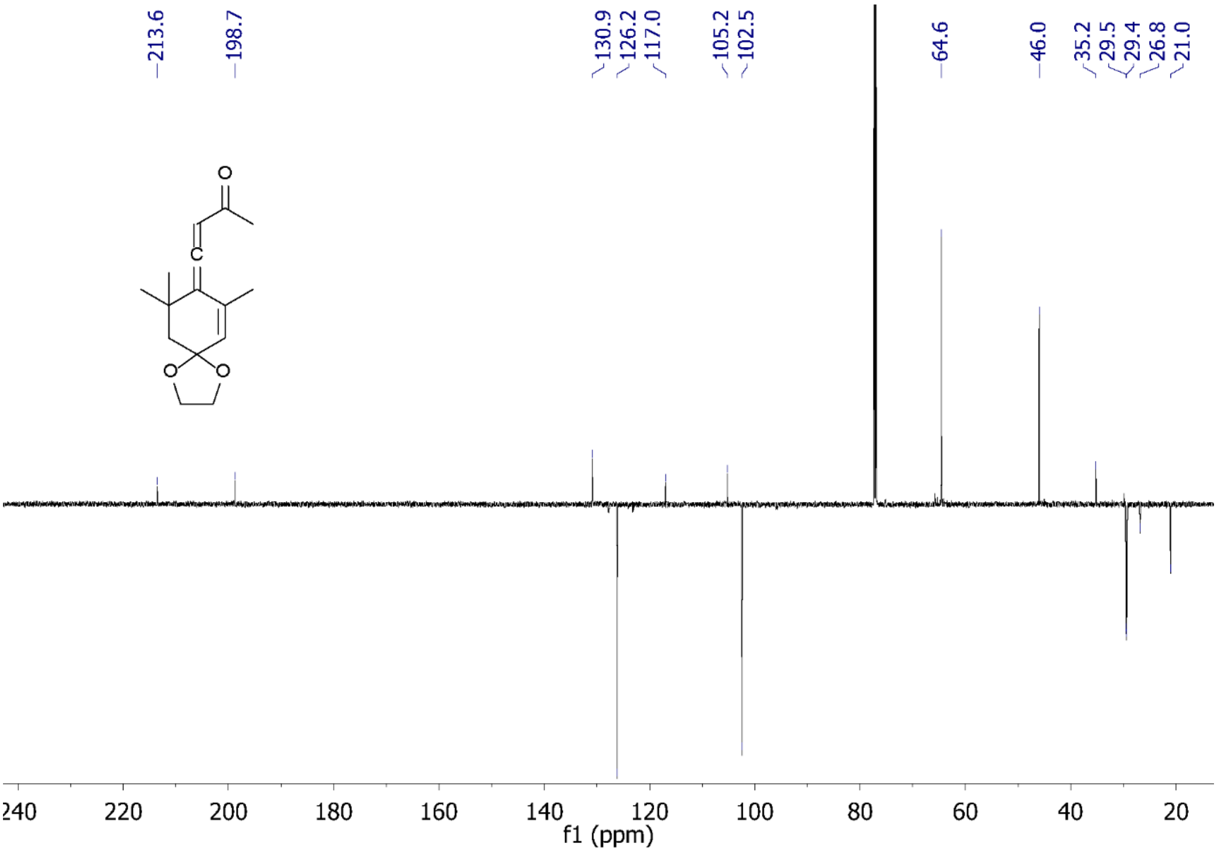

Figure S10. ^{13}C APT NMR spectrum of compounds **8a,b** (151MHz, 25°C, $CDCl_3$).

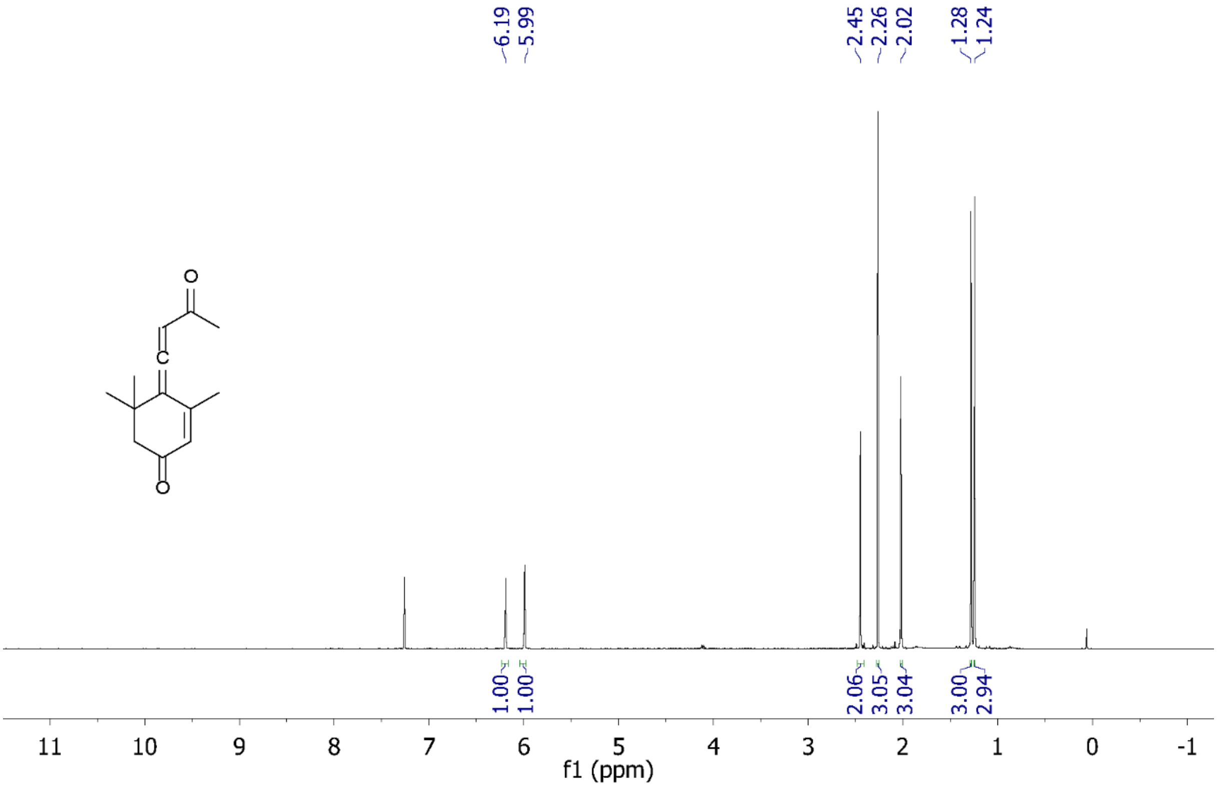

Figure S11. ^{1}H NMR spectrum of callunene (**1a,b**) (400 MHz, 25°C, $CDCl_3$).

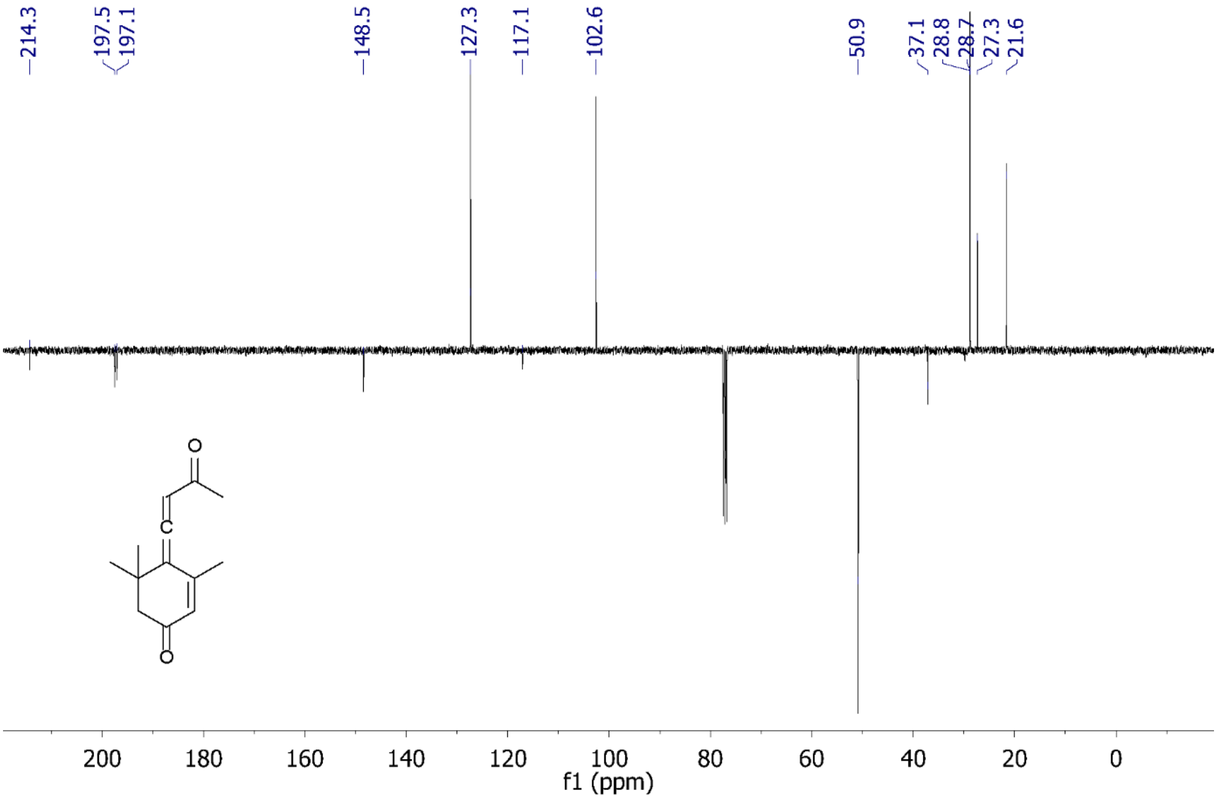

Figure S12. ^{13}C APT NMR spectrum of callunene (**1a,b**) (101 MHz, 25°C, $CDCl_3$).

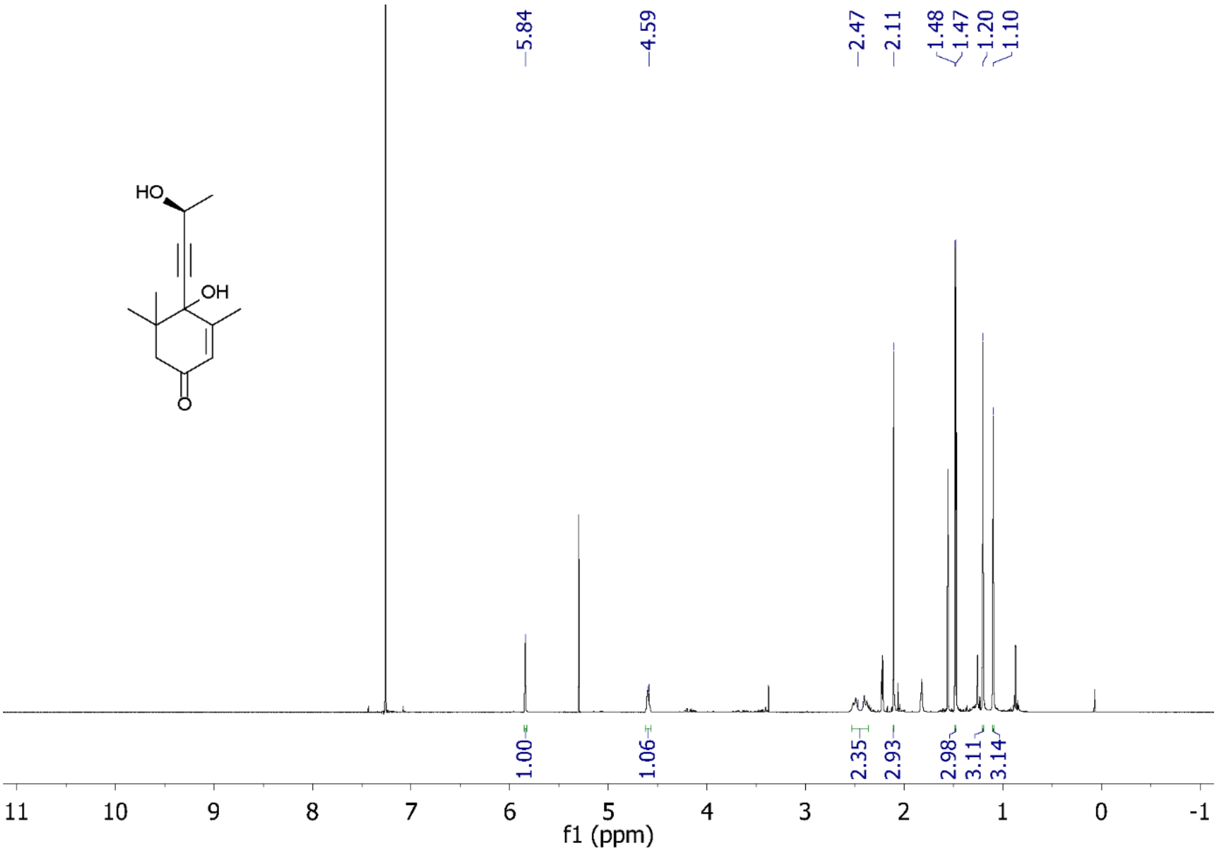

Figure S13. ^{1}H NMR spectrum of compound **S1** (600 MHz, 25°C, $CDCl_3$).

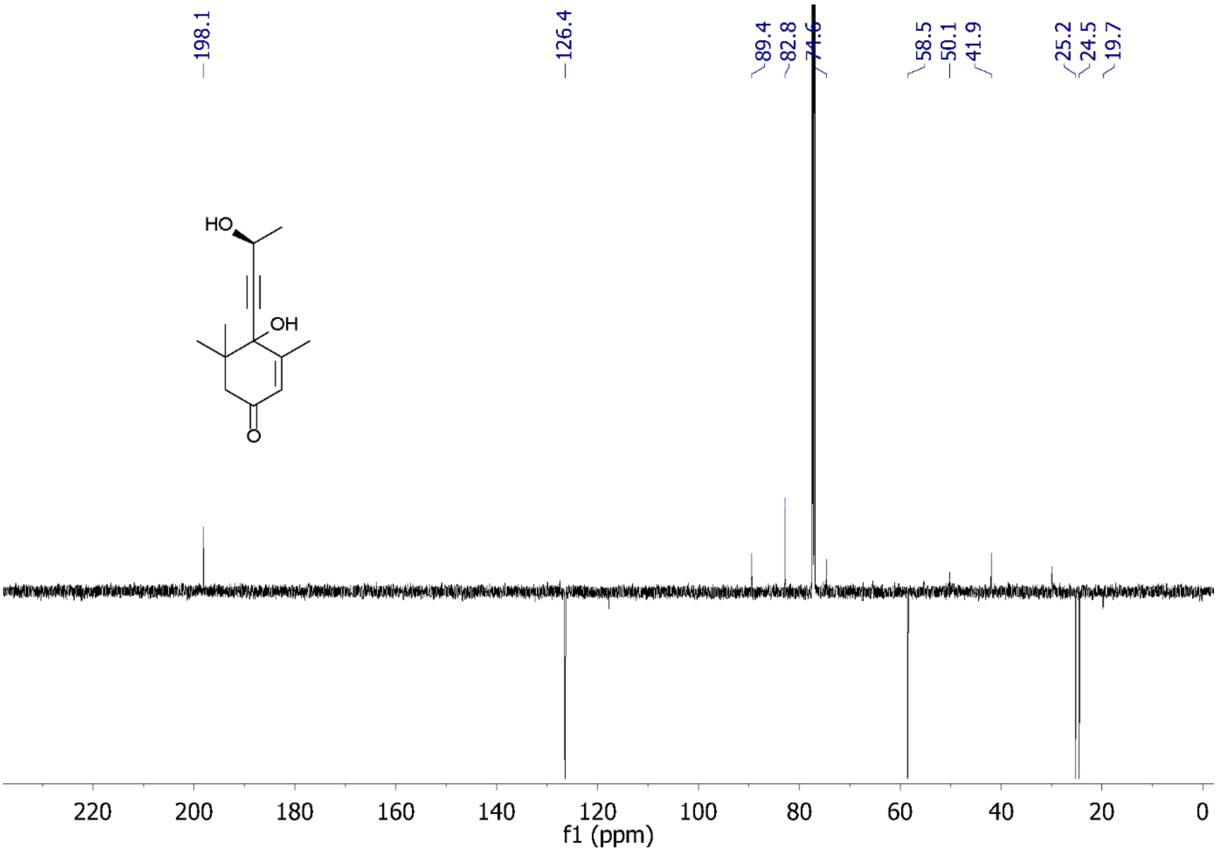

Figure S14. ^{13}C APT NMR spectrum of compound **S1** (151 MHz, 25°C, $CDCl_3$).

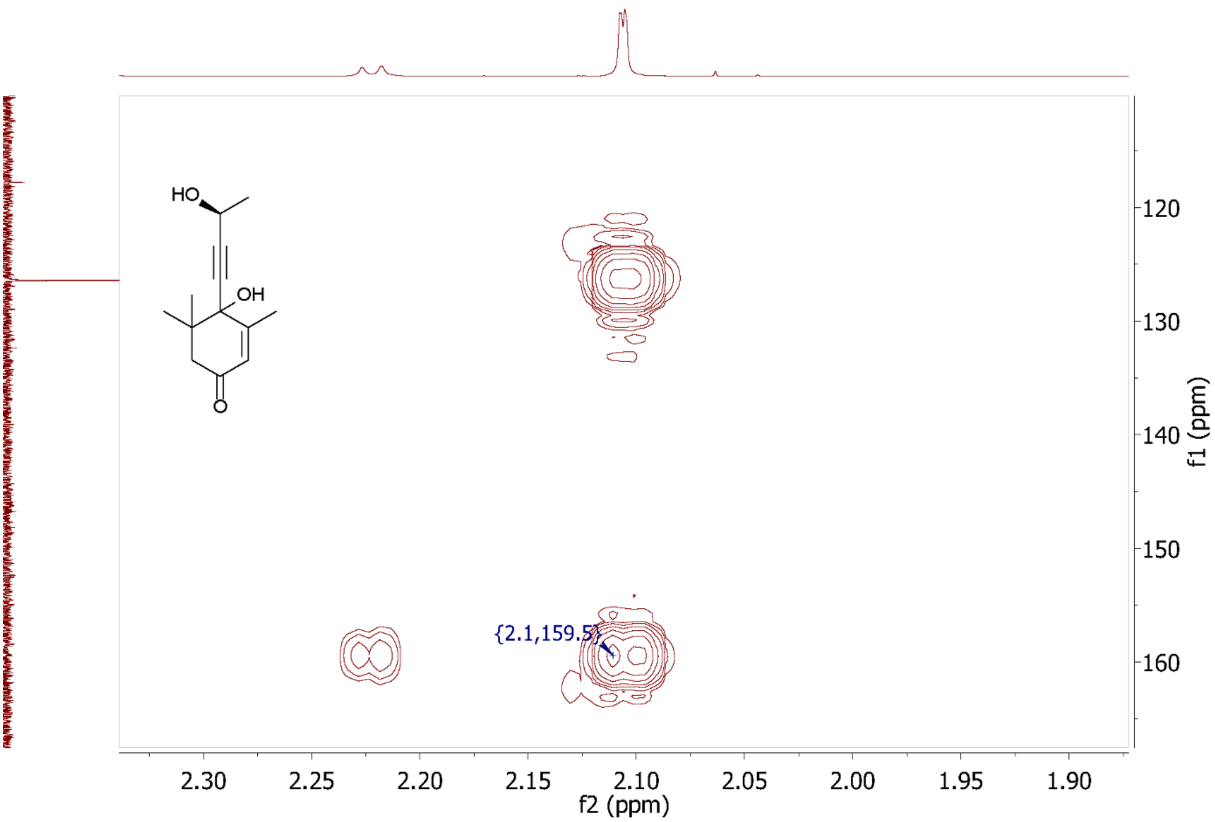

Figure S15. A part of ^{1}H- ^{13}C HMBC NMR spectra of compound **S1** showing the position of signal of C2 (600 and 151 MHz, 25°C, $CDCl_3$).

Complexation with chiral shifting agents

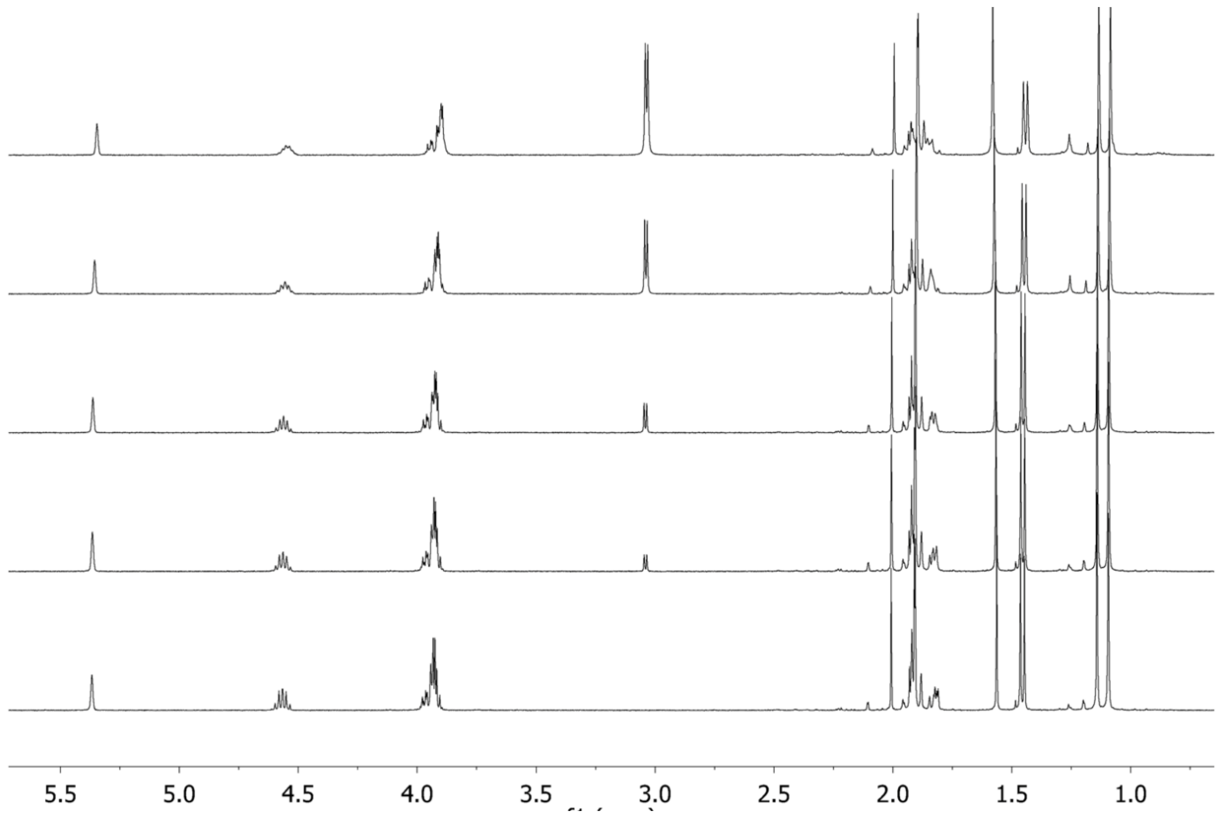

Figure S16. Enantioseparation of stereoisomers **6a-d** with Pirkle's alcohol (**A**) at 8 μM concentration. Bottom to top 0, 0.5, 1, 3, 6 equiv, ^{1}H NMR, 400 MHz, 25 °C, $CDCl_3$. (no changes).

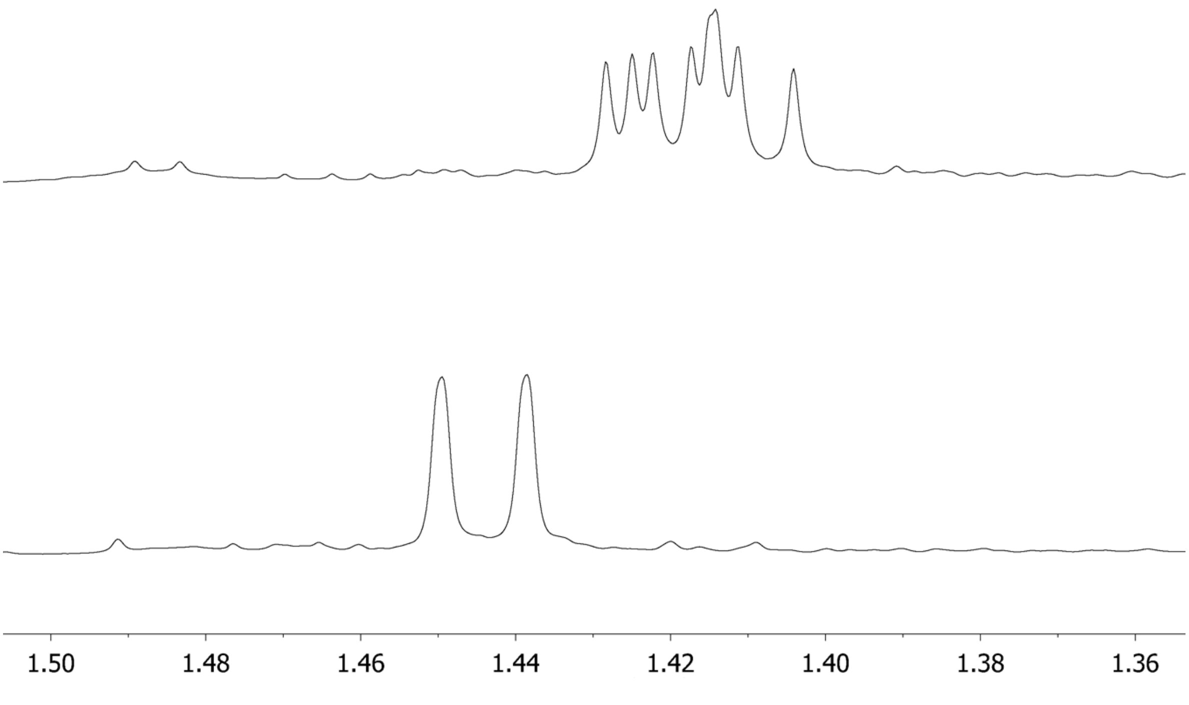

Figure S17. Enantioseparation of stereoisomers **6a-d** with Pirkle's alcohol (**A**) at 0.1 M concentration, zoomed methyl signal at position 4'. Bottom to top 0, 12 equiv, ^{1}H NMR, 400 MHz, 25 °C, $CDCl_3$.

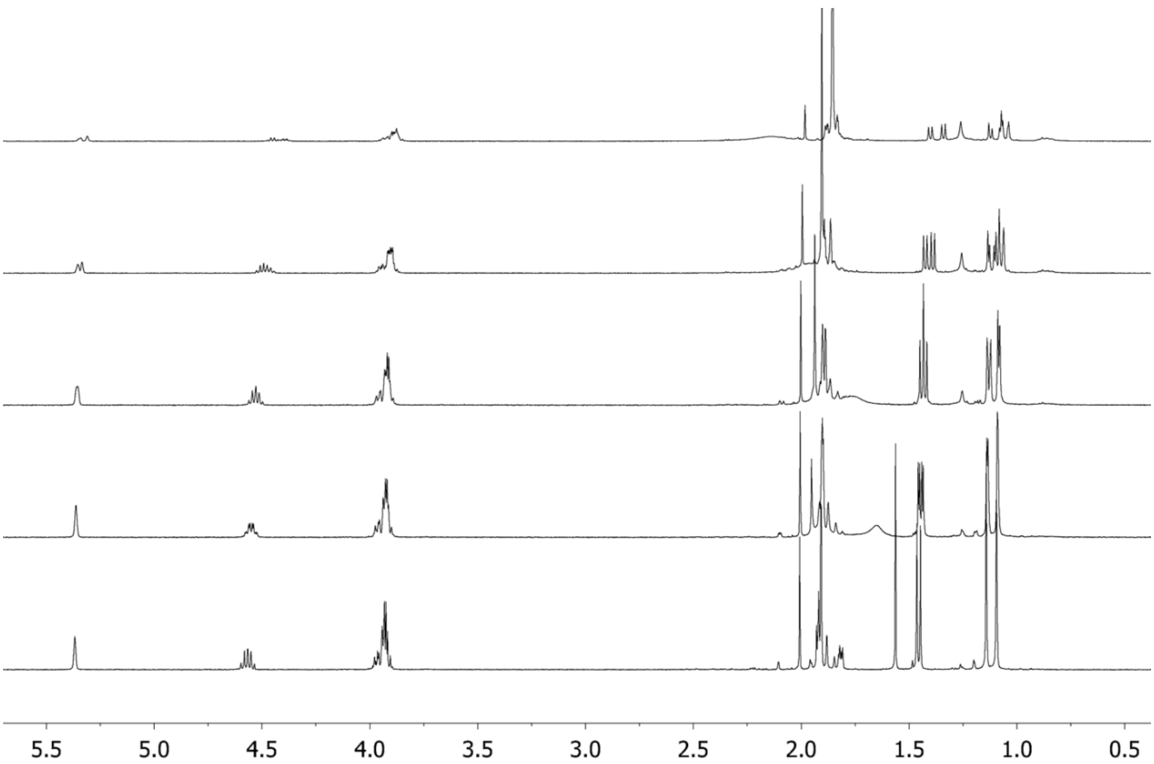

Figure S18. Enantioseparation of stereoisomers **6a-d** with reagent **B** at 8 µM concentration. Bottom to top 0, 0.5, 1, 3, 6 equiv, ^{1}H NMR, 400 MHz, 25 °C, $CDCl_3$. (two sets of enantiomers visible, no recognition of diastereomers).

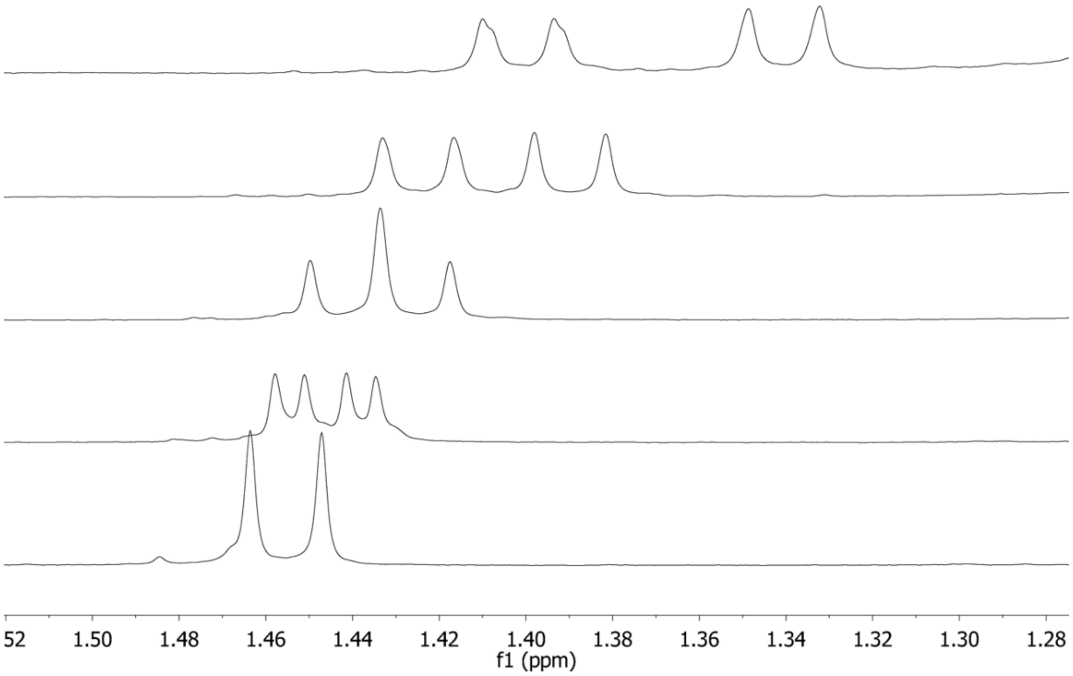

Figure S19. Zoom of the 4' methyl signal region for enantioseparation of stereoisomers **6a-d** with reagent **B** at 8 µM concentration. Bottom to top 0, 0.5, 1, 3, 6 equiv, ^{1}H NMR, 400 MHz, 25 °C, $CDCl_3$. (two sets of enantiomers visible, no recognition of diastereomers).

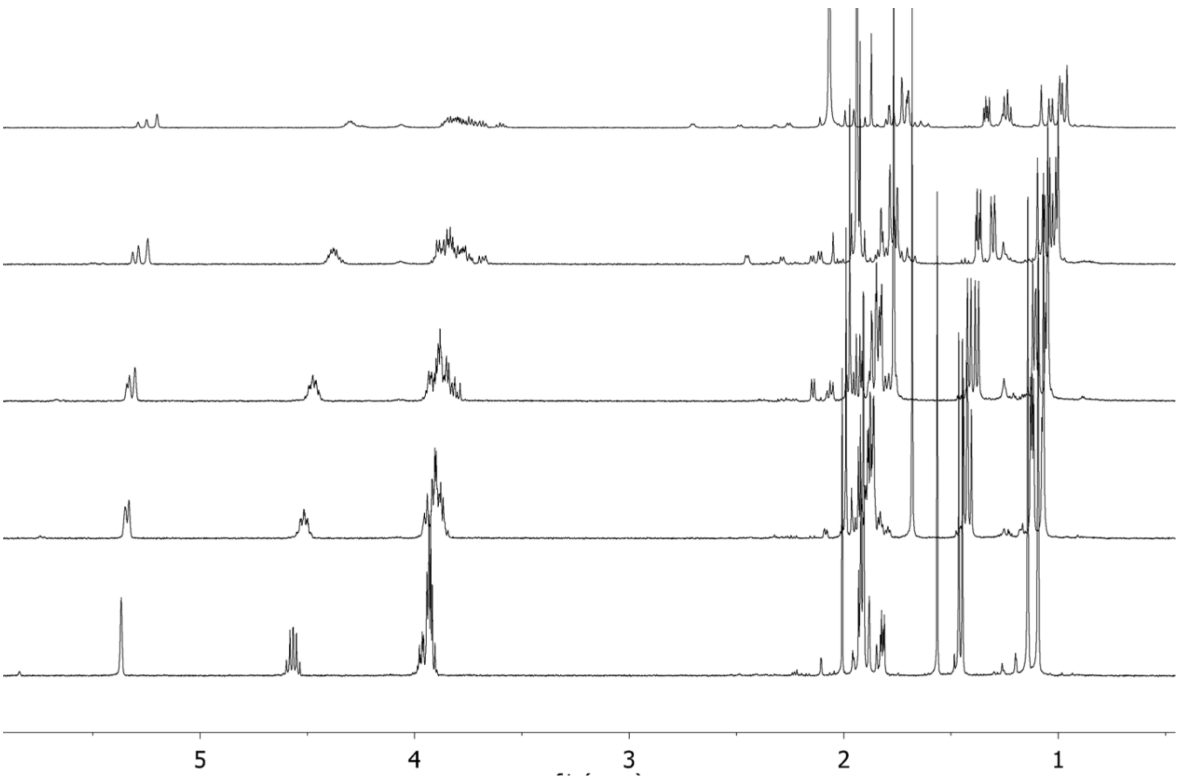

Figure S20. Enantioseparation of stereoisomers **6a-d** with reagent **C** at 8 μM concentration. Bottom to top 0, 0.5, 1, 3, 6 equiv, ^{1}H NMR, 400 MHz, 25 °C, $CDCl_3$.

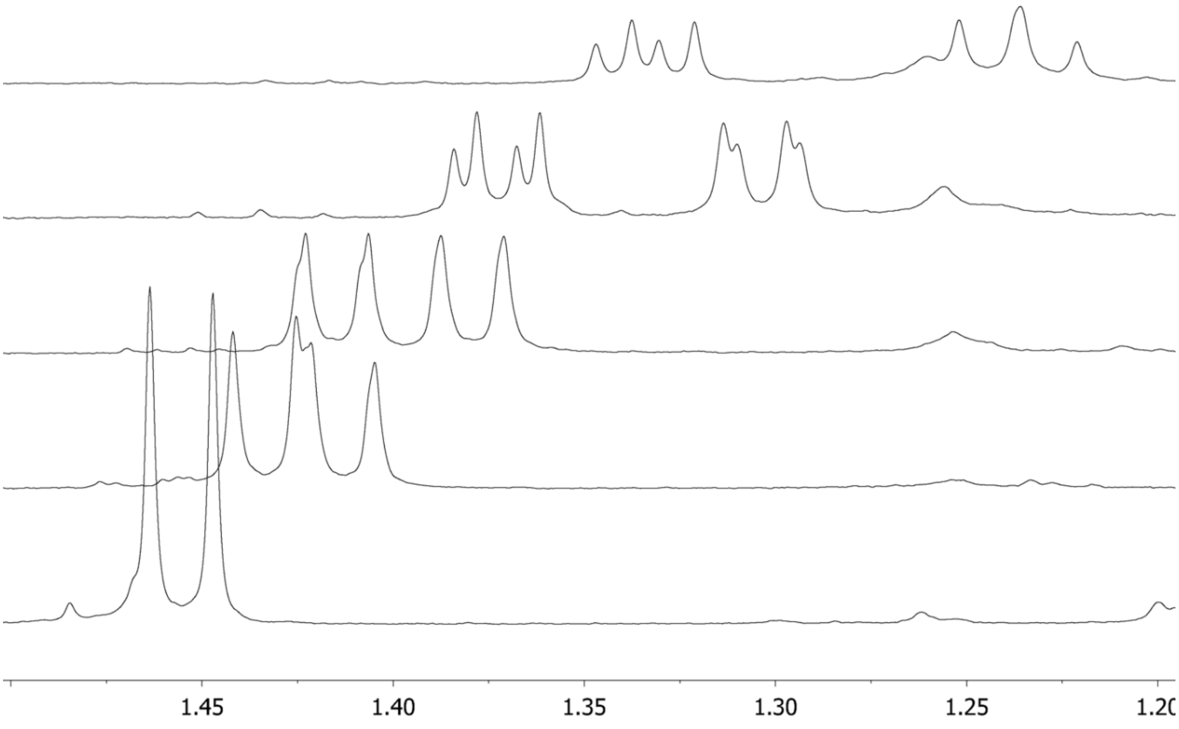

Figure S21. Zoom of the 4’ methyl signal region for enantioseparation of stereoisomers **6a-d** with reagent **C** at 8 μM concentration. Bottom to top 0, 0.5, 1, 3, 6 equiv, ^{1}H NMR, 400 MHz, 25 °C, $CDCl_3$. (from 3 equiv separation of diastereomers visible, showing unequal distribution of diastereomers).

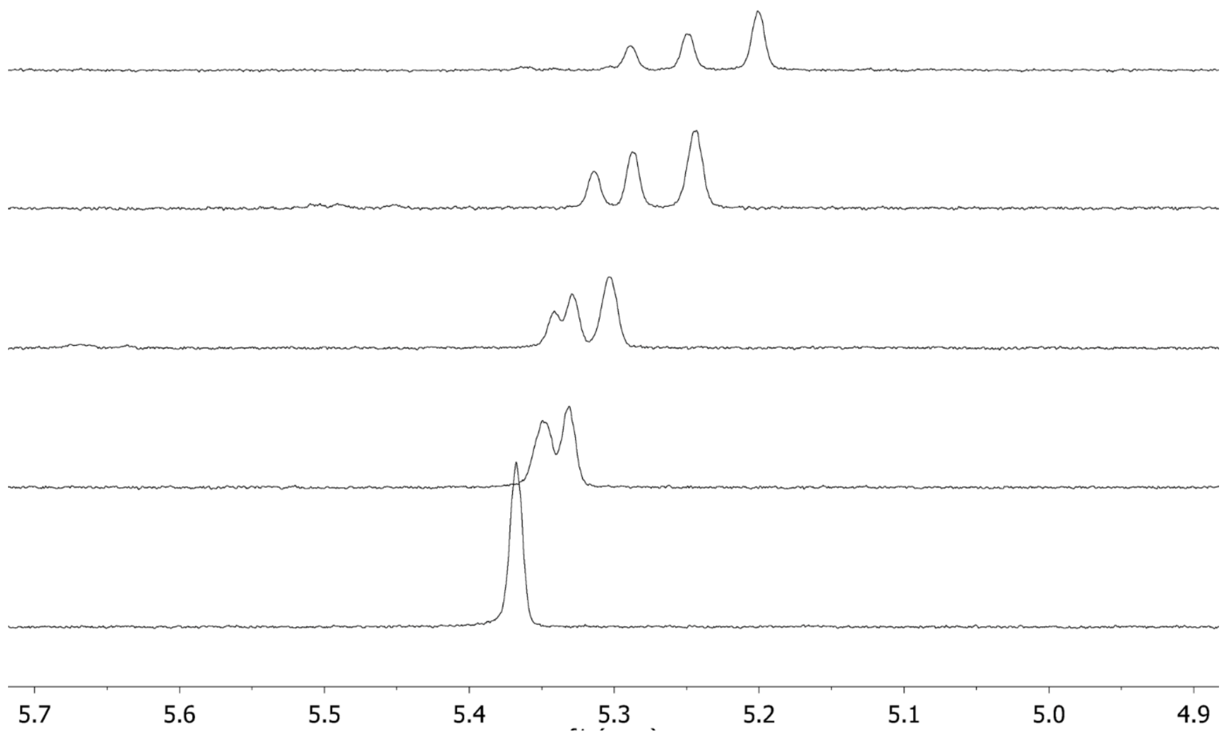

Figure S22. Zoom of the 2-hydrogen signal region for enantioseparation of stereoisomers **6a-d** with reagent **C** at 8 μM concentration. Bottom to top 0, 0.5, 1, 3, 6 equiv, ^{1}H NMR, 400 MHz, 25 °C, $CDCl_3$. (from 3 equiv separation of diastereomers visible, showing unequal distribution of diastereomers).

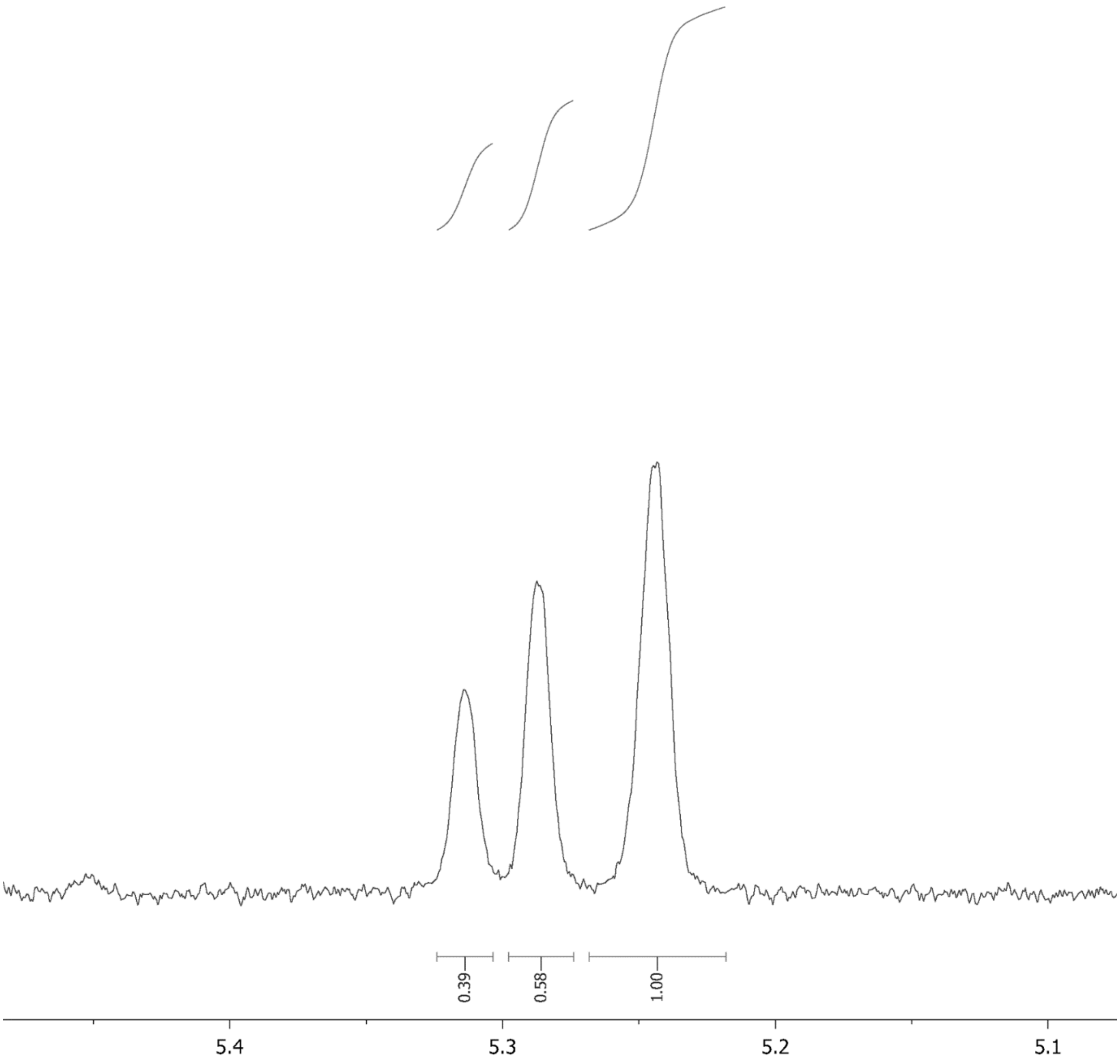

Figure S23. Zoom of the 2-hydrogen signal region for enantioseparation of stereoisomers **6a-d** with reagent **C** at 8 μM concentration at 6 equiv of **C**, ^{1}H NMR, 400 MHz, 25 °C, $CDCl_3$. (2:3 distribution of diastereomers).

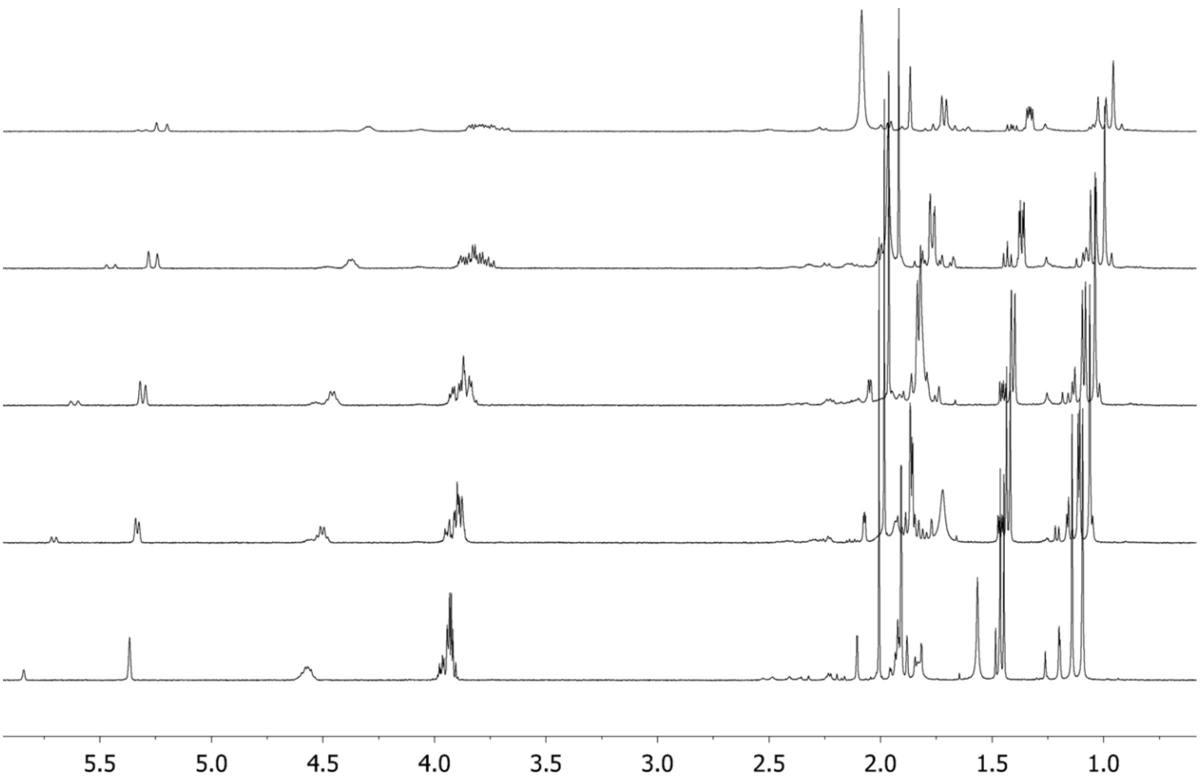

Figure S24. Enantioseparation of **6a,b** with reagent **C** at 8 μM concentration. Bottom to top 0, 0.5, 1, 3, 6 equiv, ^{1}H NMR, 400 MHz, 25 °C, $CDCl_3$. (product of deprotection, **S1**, detected).

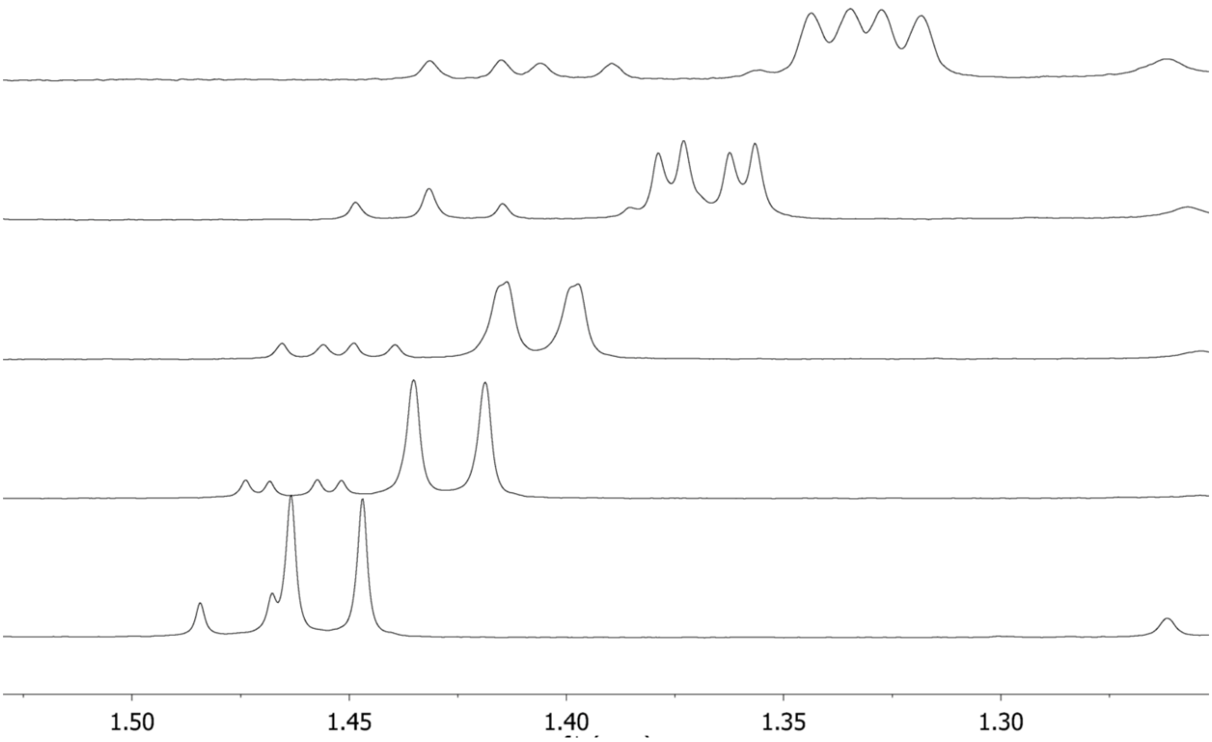

Figure S25. Zoom of the 4’ methyl signal region for enantioseparation of **6a,b** with reagent **C** at 8 μM concentration. Bottom to top 0, 0.5, 1, 3, 6 equiv, ^{1}H NMR, 400 MHz, 25 °C, $CDCl_3$. (product of deprotection **S1** visible).

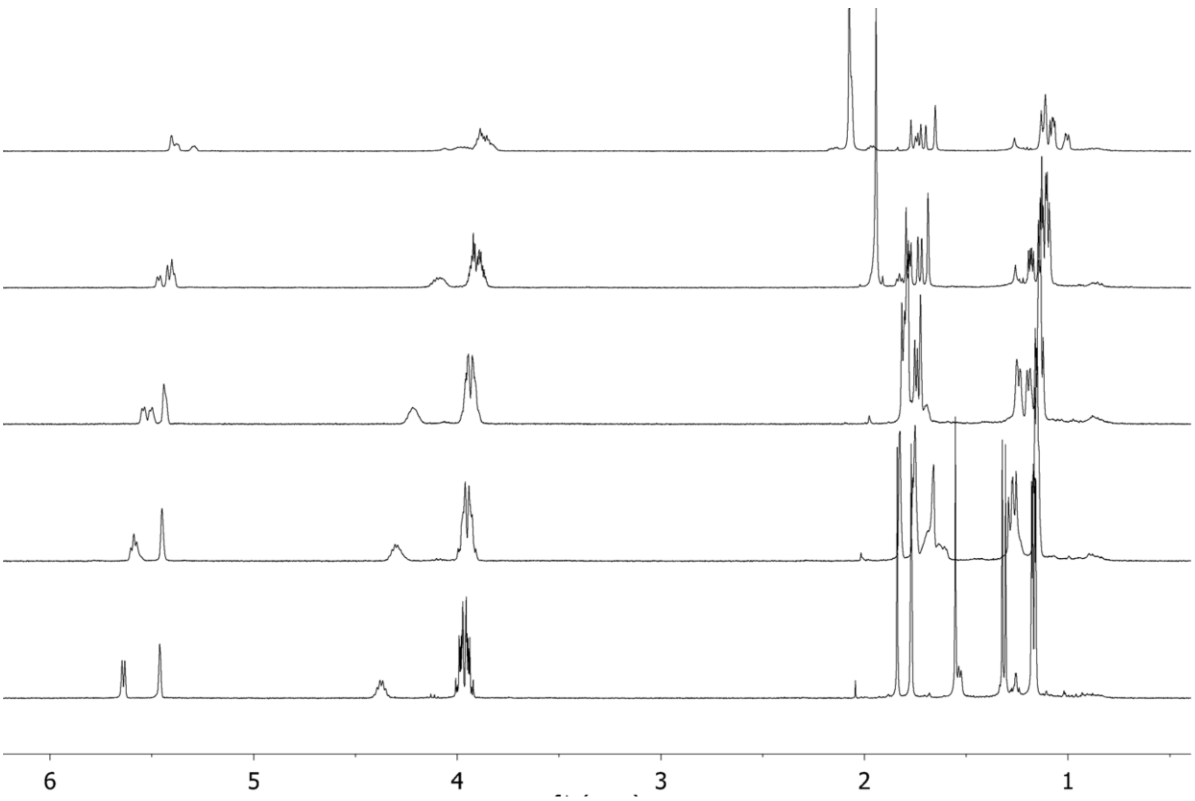

Figure S26. Enantioseparation of **7a-d** with reagent **C** at 8 µM concentration. Bottom to top 0, 0.5, 1, 3, 6 equiv, ^{1}H NMR, 400 MHz, 25 °C, $CDCl_3$. (zoomed methyl signal region available in the main text, Figure 3).

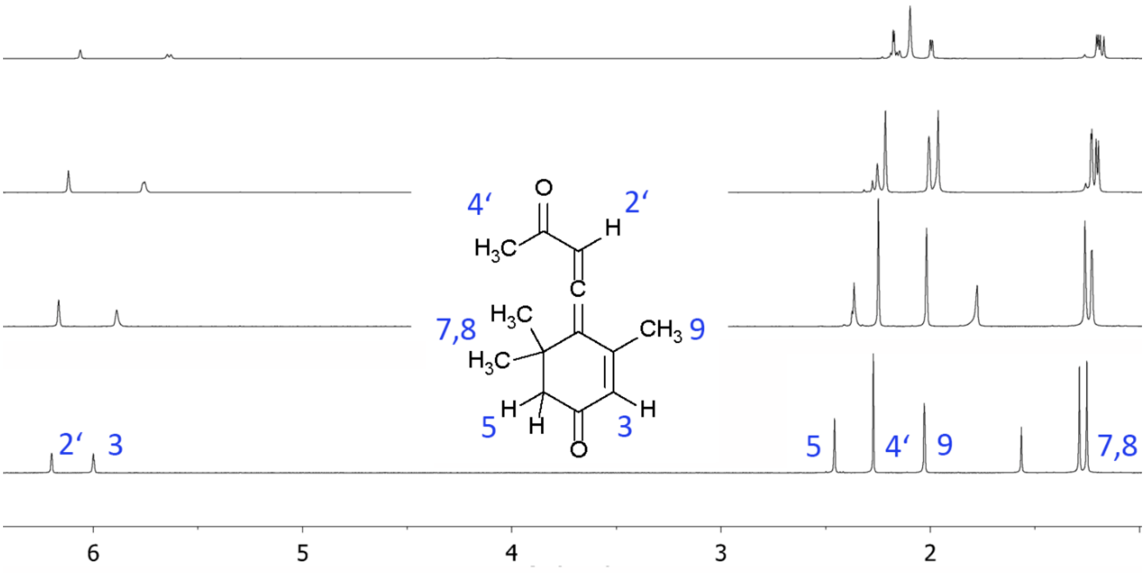

Figure S27. Enantioseparation of synthetic callunene (**1a,b**) with reagent **C** at 8 µM concentration. Bottom to top 0, 1, 3, 6 equiv, ^{1}H NMR, 400 MHz, 25 °C, $CDCl_3$. (zoomed regions available in the main text, Figure 4).

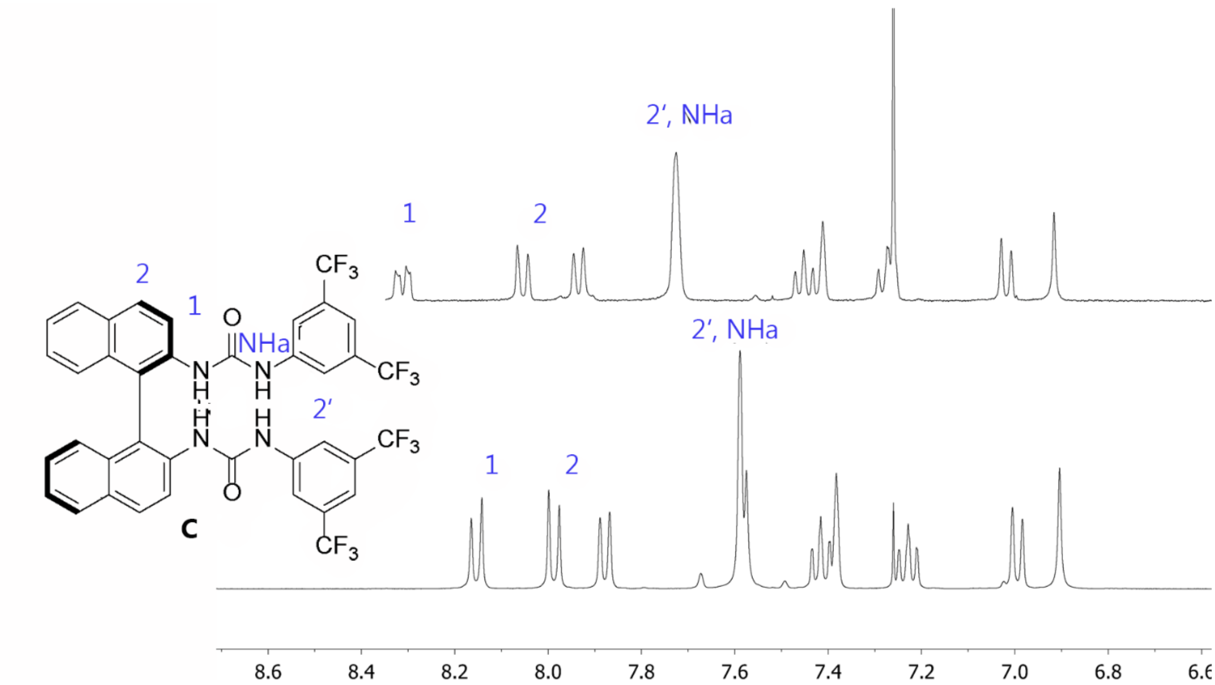

Figure S28. Enantioseparation of synthetic callunene (**1a,b**) with reagent **C**, signals of reagent **C**. Bottom to top 6, 0.5 equiv to callunene, ^{1}H NMR, 400 MHz, 25 °C, $CDCl_3$.

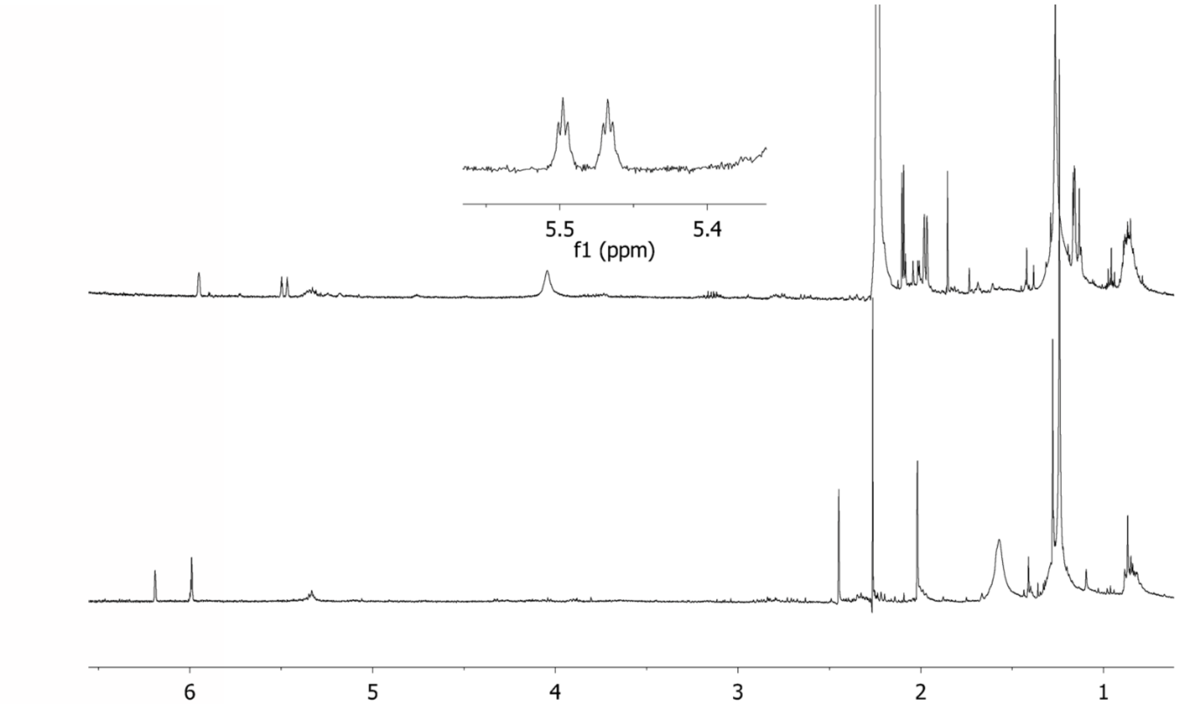

Figure S29. Enantioseparation of natural callunene with reagent **C** at 2.7 mg/0.5 mL of $CDCl_3$. Bottom to top 0, 6 equiv, ^{1}H NMR, 400 MHz, 25 °C, $CDCl_3$, signals of proton 3 zoomed.

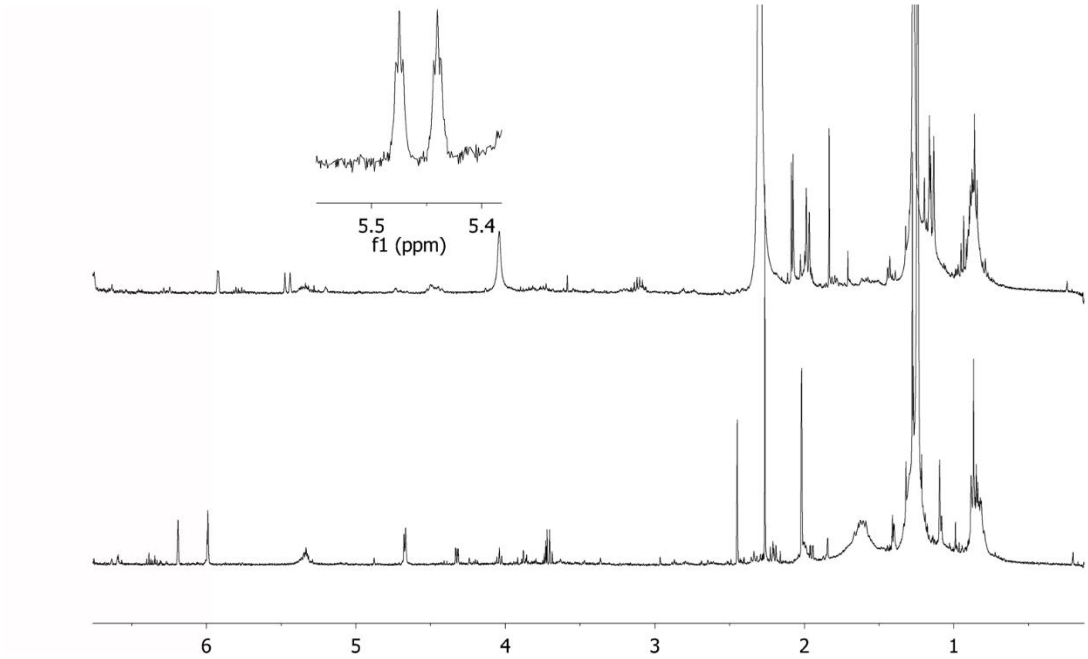

Figure S30. Enantioseparation of natural callunene with reagent **C** at 4 mg/0.5 mL of $CDCl_3$. Bottom to top 0, 6 equiv, ^1H NMR, 400 MHz, 25 °C, $CDCl_3$, signals of proton 3 zoomed.

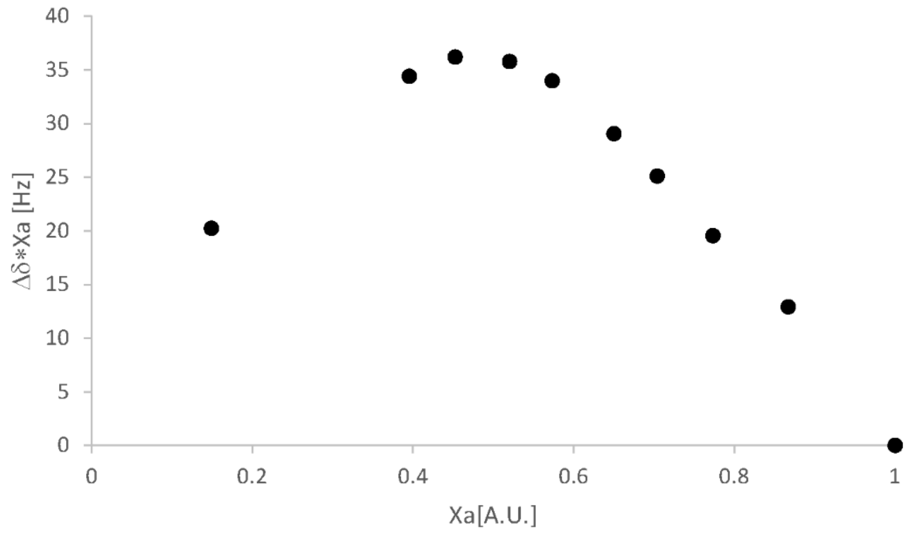

Figure S31. Job plot for the complex of **C** with **1a,b**. (^1H NMR, 400 MHz, 25 °C, $CDCl_3$, measured at concentration of **1a,b** of 1.5×10^{-2} M.)

Calculated complex structures

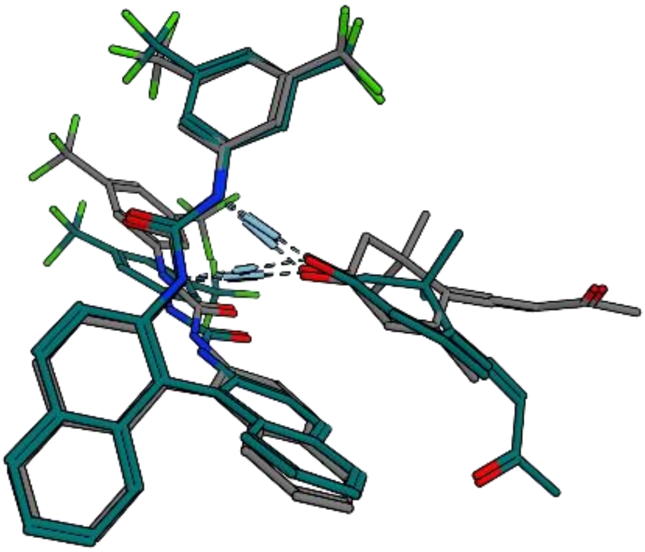

Figure S32. Calculated structures of enantiomers **1a** and **1b** in the complex with reagent **C,** overlapped. Protons in the positions 3 and 5 of R_a-callunene (grey) are in closer proximity to the reagent **C**.

HPLC separation

Table S1. Screening of conditions for HPLC separation of callunene enantiomers **1a** and **1b**. (IPA- propan-2-ol, EtOH- ethanol, DEA- diethylamine, FA- formic acid); R_FS – retention time of S_a-enantiomer, ΔR_F – retention time difference of enantiomers, dW/½ - width of the peak in the half of its height.

Column	Mobile phase composition									dW/½ [min]	
Lux i-amylose	Hexane [%]	Heptane [%]	IPA [%]	EtOH [%]	DEA [%]	FA [%]	Cooling to 5 °C	R_F S [min]	ΔR_F [min]	S	R
5	97.45		2.5		0.05			19.5	1	0.8	0.8
5	97.45		2.5		0.05		yes	37.5	2	1.5	1.5
5	94.90		5.0		0.10			27.5	0	1.5	1.5
3		97.45	2.5		0.05			24.8	1.2	1	1
3		98.95	1.0		0.05			65	0	1.5	1.5
3	94.95			5	0.05			20.5	2.3	1.2	1.2
3	94.90			5	0.10	0.1		14.8	1.5	0.5	0.6
3	94.90			5	0.10	0.1	yes	21.2	4	0.5	1.8
3		94.90	5.0		0.10	0.1		14.5	2	0.4	0.5
3		94.90	5.0		0.10	0.1	yes	16	2	0.4	0.5

For mobile phase screening, Agilent 1100 series HPLC system (Waldbron, Germany) was used.

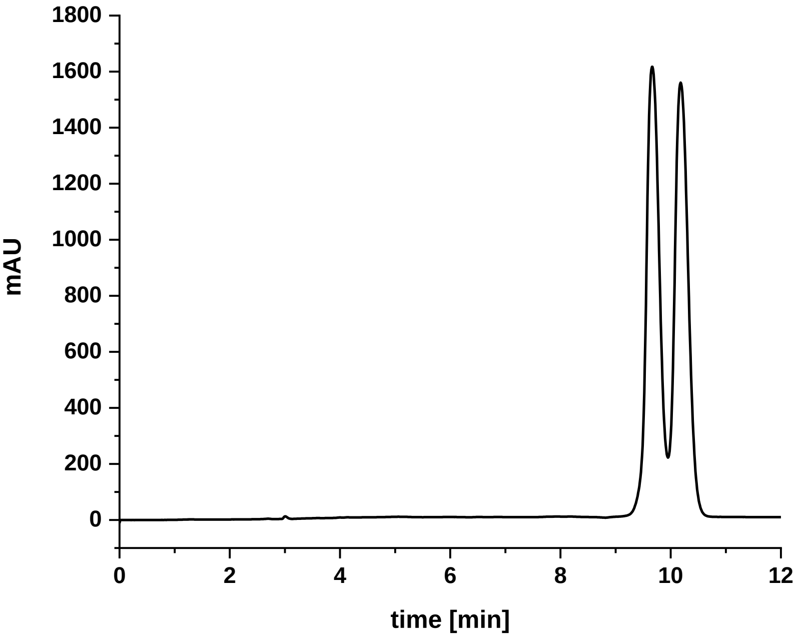

Figure S33. Chiral resolution of callunene enantiomers (**1a,b**) on Lux i-Amylose-3 column (250×4.6 mm ID, 5 µm) in a mobile phase composed of heptane/propan-2-ol (95/5, v/v) with formic acid (0.1%) and diethylamine (0.1%) at a flow rate of 1 mL min^{-1} at 20 °C and the detection wavelength of 254 nm. The concentration of the sample was 5 mg mL^{-1}. The peaks were manually collected after the detector using 6 cm long capillary with 0.018 mm in diameter.

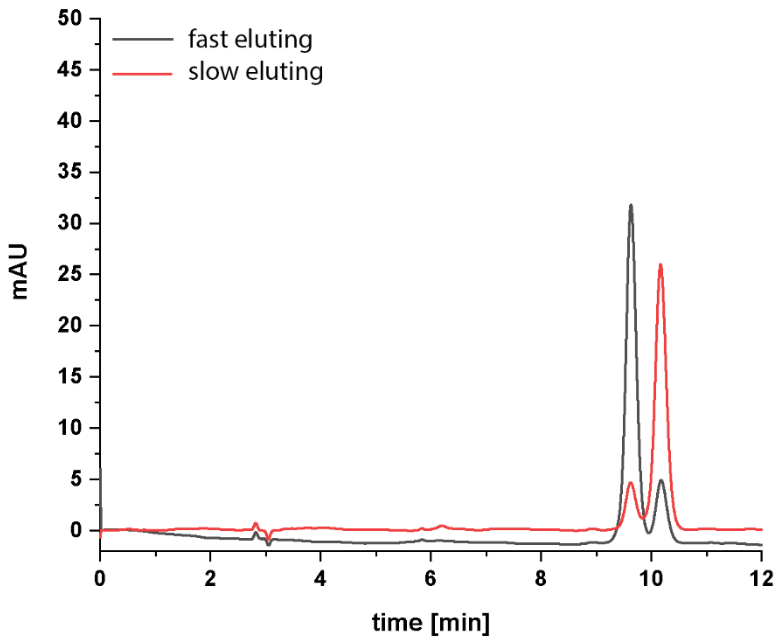

Figure S34. The purity of manually collected peaks of callunene enantiomers (**1a,b**) was determined to be 85% and 84% for the first enantiomer and second enantiomer, respectively, which was sufficient for ECD.

DFT geometry optimization

Table S2. Energies for the conformers of *S*$_a$-callunene (**1a**)**,** as obtained by GFN-xTB conformational sampling.

**Conf. ID**	**E$_{tot}$ [kJ/mol]**
1	-44.06535
2	-44.06523
3	-44.06517
4	-44.06511

Table S3. Energies and Boltzmann populations at 298.15 K and 1 atm in heptane for the conformers of *S*$_a$-callunene **(1a),** as obtained by TD-DFT calculation (mpw1pw91/cc-pvtz).

**Conf. ID**	***G* [Ha]**	**Δ*G* [kcal/mol]**	***χ*$_G$ [%]**
3	-655.4853001	0.00	44.32
2	-655.4851361	0.10	37.24
1	-655.4840124	0.81	11.32
4	-655.4835773	1.08	7.13

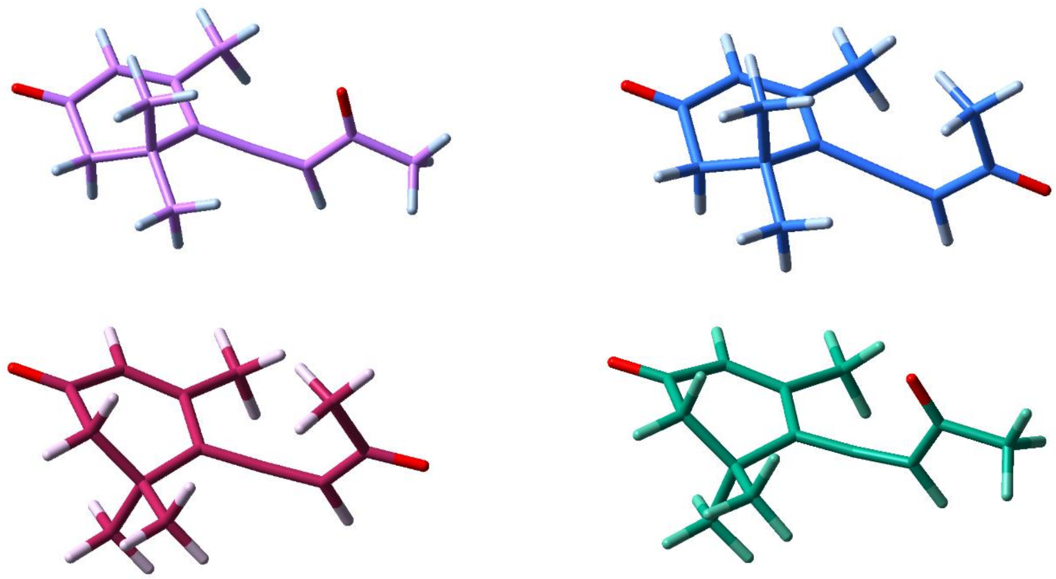

Figure S35. Optimized DFT geometries for the four conformers of S_a-callunene (**1a**). Carbons color codes per conformer id are: 1 (pink), 2 (blue), 3 (red), 4 (green).

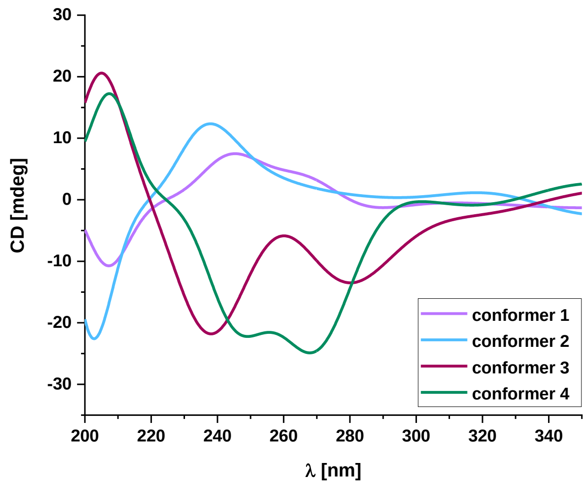

Figure S36. Theoretical ECD spectra for the four conformers of S_a-callunene (**1a**) (mpw1pw91/cc-pvtz).
